# A Constraint-Based Orbital-Optimized Excited State Method (COOX)

**DOI:** 10.1021/acs.jctc.4c00467

**Published:** 2024-09-30

**Authors:** Jörg Kussmann, Yannick Lemke, Anthea Weinbrenner, Christian Ochsenfeld

**Affiliations:** † Chair of Theoretical Chemistry, Department of Chemistry, Ludwig-Maximilians-Universität in Munich (LMU), München D-81377, Germany; ‡ Max-Planck-Institute for Solid State Research, Stuttgart D-70659, Germany

## Abstract

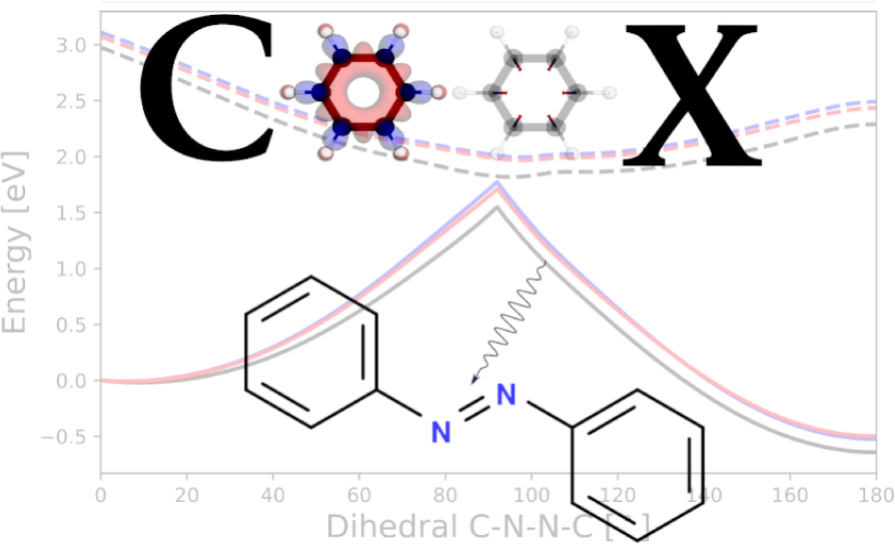

In this work, we present a novel method to directly calculate targeted electronic excited states within a self-consistent field calculation based on constrained density functional theory (cDFT). The constraint is constructed from the static occupied-occupied and virtual-virtual parts of the excited state difference density from (simplified) linear-response time-dependent density functional theory calculations (LR-TDDFT). Our new method shows a stable convergence behavior, provides an accurate excited state density adhering to the Aufbau principle, and can be solved within a restricted SCF for singlet excitations to avoid spin contamination. This also allows the straightforward application of post-SCF electron-correlation methods like MP2 or direct RPA methods. We present the details of our constraint-based orbital-optimized excited state method (COOX) and compare it to similar schemes. The accuracy of excitation energies will be analyzed for a benchmark of systems, while the quality of the resulting excited state densities is investigated by evaluating excited state nuclear forces and excited state structure optimizations. We also investigate the performance of the proposed COOX method for long-range charge transfer excitations and conical intersections with the ground-state.

## 1 Introduction

The investigation of electronic excited states is crucial to the understanding of photoinduced chemical processes and the design of energy materials like solar cells, photocatalysts, or light-emitting diodes (LED). Thus, the development of efficient and accurate *ab initio* methods to determine electronic excitations is highly important to enable the routine analysis of photoactive systems by calculating, e.g., UV-spectra or simulate photoinduced dynamic processes by nonadiabatic molecular dynamics simulations (NAMD).^1−4^ Nowadays many theoretical models to determine excited states and their properties are available,^5^ although the most accurate methods like second-order coupled cluster (CC2),^6^ algebraic diagrammatic construction (ADC),^7^ complete-active-space perturbation theory (CAS-PT),^8,9^ or equation-of-motion coupled cluster methods (EOM-CC)^10−12^ are strongly limited in their range of applicability due to their significant computational costs. Therefore, adiabatic linear-response time-dependent density functional theory (LR-TDDFT)^13−18^ has emerged as the main workhorse for excited state simulations due to its relatively low computational cost and overall good accuracy.^19^ However, TDDFT suffers from some well-known shortcomings like the restriction to single excitations^20,21^ or the poor description of charge-transfer excitations.^22−25^ While the first results from the linear-response ansatz, the latter is related to derivative discontinuities and self-interaction error^26,27^ of approximate density functionals.

In recent years there is an increased interest in orbital-optimized TDDFT (OO-TDDFT) methods like ΔSCF,^28,29^ the maximum overlap method (MOM/iMOM),^30,31^ or the state-targeted energy projection method (STEP)^32^ as a promising pathway to overcome at least some of the issues of LR-TDDFT. The ΔSCF method, for example, has been shown to be able to describe double excitations and accurate charge transfer excitation energies.^33^ Furthermore, the description of conical intersections with the ground-state is also improved compared to LR-TDDFT which has been investigated for the azobenzene isomerization.^34,35^ Some notable issues of these OO-TDDFT methods is the requirement of clear-cut orbital–orbital transition for the targeted excitation, the violation of the Aufbau principle, as well as the instability of the SCF procedure that often results in a collapse back to the ground-state.^36^ Note that the collapse to the ground-state in ΔSCF calculations can be avoided by the square gradient minimization method (SGM).^37^

A possible alternative to these ΔSCF-type of methods is given by the constrained DFT (cDFT) method. Since the first formulation of cDFT by Dederichs et al.,^38^ the works by van Voorhis and coworkers^39−41^ led to a revived interest in cDFT for a wide range of applications.^42−48^ With respect to excited states, the description of intermolecular charge-transfer excitations lends itself naturally to cDFT by enforcing the transition of a single electron from donor to acceptor molecule. Since these type of excitations can be described by the interaction of ionized ground-states, a self-consistent ground-state method like cDFT strongly improves on the results of LR-TDDFT.

Recently, two different cDFT methods have been proposed that aim at describing electronic excitations for both long-range charge-transfer and small radius Frenkel excitations, the excited cDFT (x-cDFT)^49^ and the transition cDFT (t-cDFT)^50^ methods. In contrast to ΔSCF, these cDFT-based methods have the advantage that the resulting wave function follows the Aufbau principle and also should prevent the collapse to the ground-state. While first results for these methods are promising, x-cDFT and t-cDFT both suffer from some issues, especially the range of applicability as well as the quality of the excited state density as will be discussed in the following sections.

Therefore, we propose a different orbital-optimized excited state method based on cDFT, the constraint-based orbital-optimized excitations (COOX) method, which strongly improves on the results of the aforementioned methods.

In the next section the general cDFT equations as well as the x-cDFT and t-cDFT methods will be briefly reviewed, followed by the detailed description of our new COOX method. The accuracy of our ansatz will be analyzed and compared to the other methods in the final section. Apart from energy benchmarks we will also discuss its performance for specific problems like long-range charge transfer excitations and conical intersections.

## 2 Theory

Following the formulation by van Voorhis and coworkers,^41,51^ we define an energy functional considering an additional constraint potential **W**_*c*_,
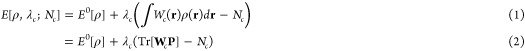
1with the regular Kohn–Sham energy functional , a Lagrangian multiplier λ_*c*_, the one-electron density matrix **P**, and a constant parameter *N*_*c*_. As shown in ref (51), the cDFT stationary point is a minimum with respect to the density ρ and a maximum with respect to λ_*c*_
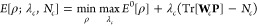
3Thus, the constrained SCF optimization procedure can be described by a nested-loop algorithm that requires only slight modifications to already existing regular SCF codes. Here, in each SCF cycle (macro iteration/minimization) an optimization step to fulfill the constraint condition is embedded (micro iteration/maximization).

The objective of the constraint optimization is usually to enforce a redistribution of the electronic charge or magnetization by employing (local) potentials^41^ that can be constructed from spatial subspace projectors of Mulliken,^52^ Löwdin,^53^ Hirshfeld,^54^ or Becke type.^55^

The use of cDFT to describe electronic excitations comes natural for, e.g., intermolecular charge-transfer excitations that can be directly described by a constraint that shifts a single electron from the donor to the acceptor.^40^ In contrast, local Frenkel excitations or higher excitations cannot be described by simple spatial constraints and require a different approach. Recently, different cDFT methods to determine arbitrary excited states have been proposed, the x-cDFT by Ramos and Pavanello^49^ and the t-cDFT method by Stella et al.^50^

The x-cDFT method is limited to the evaluation of the lowest excited state only, where a single constraint is employed to enforce the transition of a single electron from the occupied to the virtual MO subspace. The constraint potential is given by the (covariant) virtual density matrix:
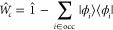
4
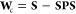
5with the one-electron density matrix **P** and the metric **S**. To ensure a single electron transition, the constraint is applied to the α-electron density only within an unrestricted constrained SCF calculation and the parameter *N*_*c*_ is set to 1. As discussed in ref. (49), the derivative of the energy with respect to the number of electrons undergoing the transition (*N*_*c*_) equates to the work necessary to force an electron from the occupied to the virtual MO space:
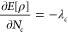
6Thus, −λ_*c*_ can also be interpreted as the excitation energy and has been shown by the authors to provide on average better results based on their chosen benchmark set.^49^

The t-cDFT method,^50^ in contrast, should be able to determine arbitrary (higher) excitations by employing constraint potentials obtained from single, symmetrized transition densities (dyadic product of two MO-vectors) with the constant parameter *N* given by the corresponding (TDA-)TDDFT MO transition coefficient (). Thus, depending on the number of involved molecular orbitals, multiple constraints may be required:
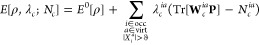
7
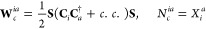
8with *i* and *a* referring to occupied and virtual MOs, respectively, ground-state MO vectors **C** and MO transition coefficients **X** as parameters . The threshold ϑ determines which transition coefficients are considered in the constraint, therefore a reduced set of constraints has to be renormalized to ensure a proper representation of a single electron excitation. Here, we will restrict our notation to TDA-TDDFT^56^ calculations, while the corresponding ansatz for TDDFT (or RPA) results is straightforward. It is obvious that t-cDFT is computationally more demanding as compared to x-cDFT due to a possibly large number of constraint terms and a preceding TDDFT calculation, but in turn t-cDFT allows to evaluate any specific excited state.

As already indicated and will also be shown in the following sections, both methods suffer from severe shortcomings that limit their range of applicability.

### 2.1 Constraint-Based Orbital-Optimized Excitations (COOX)

In order to overcome the shortcomings of not only the x-cDFT and t-cDFT methods, but also some of the ΔSCF, MOM, or STEP methods, we propose our constraint-based orbital-optimized excited state method (COOX), that similar to x-cDFT relies on a single constraint only but also draws on the MO transition coefficients like t-cDFT. Here, the constraint is given by the difference of the static parts of the excited state difference density **ΔP**, i.e., the virtual–virtual (**ΔP**^virt^) and occupied–occupied (**ΔP**^occ^) subspace projections of the difference density:
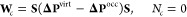
9which can be directly determined by matrix–matrix multiplications of the MO transition coefficients matrix **X**:
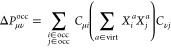
10
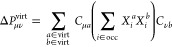
11Note that this constraint can be seen as a superposition of two constraints, i.e., the expulsion of an electron from **ΔP**^occ^ and the admittance of an electron to **ΔP**^virt^, respectively, with a parameter *N*_*c*_ equal to 1 for each constraint term. For the combined constraint in eq 9, the parameter *N*_*c*_ is therefore equal to 0. This also means that at convergence the (free) energy of the constrained system^41^ equals that of the unconstrained Hamiltonian:
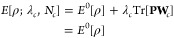
12since . This implies that the obtained electron density is also a stationary point for the unconstrained (unperturbed) Hamiltonian as it would be expected for an eigenstate, resembling a free oscillation in contrast to x-cDFT or t-cDFT, where the constraint/perturbation terms contribute to the energy similar to the impact of oscillatory electric field perturbation in the picture of a forced oscillation.^57^

A specific advantage of this approach is that for singlet excitations the constrained SCF equations can be solved in a restricted fashion, i.e., one obtains a single excited state density for α and β electrons resembling an excitation of half an α and β electron each. Thus, spin contamination is not an issue and a subsequent spin purification^58,59^ is not required.

Note that the restricted calculation requires fractional occupation numbers which are induced by applying Fermi-smearing.^60^ In some cases, e.g., for degenerate highest-occupied and lowest-unoccupied molecular orbitals (HOMO/LUMO) as for example in benzene, this can result in undesirable double-excitations. As will be shown in the next section, this case can be easily identified due to a doubling in the excitation energy. A straightforward solution in those cases is to resort to an unrestricted calculation where the COOX constraint is only applied to the α spin channel, thus resulting in a single-electron excitation.

Alternatively, another solution in those cases is to scale **ΔP**^virt^ in the constraint potential **W**_*c*_ which can be considered as employing level-shifting in the constraint calculation. Note that this approach is more preferable since it allows us to still obtain a restricted solution preventing spin contamination. We can derive the scaling factor from investigating the resulting expectation value of the constraint potential with the (relaxed) excited state density  within the linear-response framework. Therefore, let us consider the following matrices from TDA-TDDFT in an orthonormal basis:
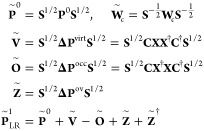
with the (unperturbed) ground-state density **P**^0^ and the occupied-virtual part **Z** of the excited state density **P**^1^. Note that the transition coefficients **X** are of dimension *N*_virt_,*N*_occ_. Thus, the MO coefficients **C** in the formation of  are restricted to virtual orbitals only and for  only occupied orbitals are used, respectively.

To fulfill the COOX constraint, the trace of  must vanish. Replacing the excited state density obtained with COOX with the one obtained from linear-response TDA-TDDFT () yields
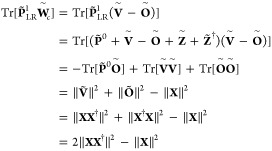
13where the Frobenius norm of **X** obtained from LR-TDDFT is always 1. One approach would be to use the value of  as the constant parameter *N*_*c*_. However, to ensure the validity of eq 12, i.e., the vanishing of the term Tr[**PW**_*c*_] at convergence, and both  and  being stationary in ρ and λ_*c*_, we can derive a factor *f*_*V*_ to form a modified constraint potential :
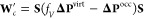
14
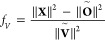
15The corresponding equations for regular TDDFT can be found in the Supporting Information. It should be stressed again that eq 13 is only valid for the excited state density obtained within the linear-response scheme, i.e., it will not be valid for the excited state density obtained with an OO-TDDFT method like COOX since the corresponding excited states will not be orthogonal to the corresponding ground-state. However, it will be shown in the following section that the proposed scaling effectively constrains the number of excited electrons to one.

In order to evaluate triplet excitations within the COOX approach, we obtain the MO transition coefficient from a restricted triplet TDDFT calculation. For the following constraint SCF calculation, a β-electron is switched to α to form a triplet state. The corresponding constraint potential is then applied to both α and β Kohn–Sham matrices with . Note that in principle a restricted open-shell calculation is possible but has so far not been investigated.

The evaluation of excited state properties, especially nuclear forces, is of particular interest as they can be treated similar to ground-state forces. Since  is stationary in ρ and λ_*c*_,^39,41,61^ the Hellmann–Feynman theorem^62,63^ can be invoked. Note that for calculations with finite basis sets the Pulay force term^64^ has to be considered. Therefore, cDFT forces only have to additionally consider the derivative of the constraint potential. Since the constraint potential in COOX is not a function of the nuclear coordinates, the implementation for conventional SCF ground-state forces can be employed. The quality of these forces—and therefore the quality of the COOX excited state electron density—will be investigated in the following section.

Our COOX method combines several features that strongly improve on other orbital-optimized excited state methods. As only a single constraint is required, the overall optimization process is stable and shows quick convergence. Since the constraint is based on the static subspace projections of the difference density, the constraint itself as well as the resulting electronic structure feature the correct symmetry. Finally, in contrast to ΔSCF-like methods, the resulting wave function follows the Aufbau principle. Thus, the regular toolset for ground-state methods can be directly applied. For example, post-SCF methods like SOS-MP2^65^ or direct RPA^66−70^ can utilize the Laplace transform ansatz, which in case of SOS-MP2 is also essential to achieve a favorable scaling behavior.^71,72^

A notable drawback considering the overall performance is the necessity to perform a TDDFT calculation to generate the constraint potentials similar to t-cDFT, while the cost of the constraint construction for x-cDFT is negligible. To overcome this potential bottleneck, the simplified TDSCF methods by Grimme^73^ can be employed. Apart from accurate excitation energies, the MO transition coefficients have been found to be of similar quality to those obtained from conventional TDDFT calculations.^74^

In the following section the results of our proposed COOX method will be compared to TDDFT, x-cDFT, and t-cDFT results for a set of molecules and excitations. Apart from the bare excitation energies, the quality of the resulting excited state electron densities will also be investigated at the example of excited state forces and structure optimizations.

## 3 Illustrative Calculations

All presented methods have been implemented in our FermiONs++ program package.^75−77^ The def2-TZVP basis^78^ has been employed in all calculations. Tight integral thresholds () and an SCF convergence threshold of  for the norm of the commutator [**F,P**]_−_ have been used throughout. Two-electron integrals were evaluated with the improved RI-Coulomb integral engine^79^ using the universal auxiliary basis “def2-universal-jfit”^80^ and the sn-LinK method.^81−83^ For the cDFT optimization step we employed the TOMS748 algorithm^84^ for x-CDFT and COOX, while a Newton–Raphson^85^ solver is used for t-cDFT. For all COOX calculations an electronic temperature of 1000 K has been chosen. For all DFT calculations the gm5 grid^86^ has been employed and the libxc,^87,88^ DFTD3,^89−91^ and gCP^92,93^ libraries have been used where required. The BFGS optimizer of the ASE package^94^ has been used for geometry optimizations. The Pymol program^95^ was used to generate depictions of molecular structures and difference density plots. While the x-cDFT method is used as described in refs. (49,96) we modified the t-cDFT method^50^ to strongly reduce the number of constraints and thus improve the convergence behavior by drawing the constraints from natural transition orbitals (NTO).^97^

We analyze the accuracy of our proposed method for a benchmark set drawn from ref (98). The quality of the resulting excited state density is investigated by calculating the nuclear derivatives of the excited state for a selection of molecules and also apply the COOX method to optimize the S_1_ geometry of biphenyl. The aforementioned problem of orbital degeneracy within the COOX scheme and its solution is discussed at the example of benzene. Finally, we investigate the quality of the COOX results for charge-transfer excitations and the description of conical intersections.

### 3.1 Excitation Energies

We evaluate singlet and triplet excitation energies for a subset of the benchmark in ref (98) with LR-TDA-TDDFT, x-cDFT, t-cDFT, and our new COOX method.

In Table 1 the singlet excitation energies are shown for different functionals and compared to the theoretical best estimates (TBE) from ref (98). The constraints for t-cDFT and COOX are formed from the corresponding transition coefficients obtained with LR-TDA-TDDFT. For the COOX calculations we give the results of the unmodified constraint (eq 9) by default, but resort to the scaled constraint (eq 14) if the deviation from a single-electron excitation character is larger than 20% (i.e., %%). Therefore, we determine the number of excited electrons by projecting the resulting excited state density **P** onto the virtual MO-space of the unperturbed ground-state
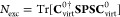
16where it should be stressed again that these states are not orthogonal due to different sets of molecular orbitals. Based on this result, we estimate the percentage of single- (%T_1_) and double-excitation (%T_2_) character of the excitation by solving:
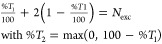
17The detailed results for both COOX versions are shown in Tables S2–S5 for all functionals in the Supporting Information.

**Table 1:** Benchmark of Singlet Excitations Energies [eV] from TDA-TDDFT Reference^a^

		PBE/def2-TZVP				PBE0/def2-TZVP				ωB97-X/def2-TZVP				HF/def2-TZVP				
Molecule	State	TDA	x-cDFT	t-cDFT	COOX	TDA	x-cDFT	t-cDFT	COOX	TDA	x-cDFT	t-cDFT	COOX	TDA	x-cDFT	t-cDFT	COOX	TBE
Ethylene	1 ^1^B_1*u*_	8.42	6.65	7.21	6.38	8.35	7.33	6.75	7.55	8.26	7.63	6.78	8.59	7.97	9.15	6.99	8.28	7.80
Butadiene	2 ^1^A_*g*_	6.31		6.06	4.34	7.04		6.04	5.97	7.83		5.36	7.26	8.32		6.56	7.19	6.55
	1 ^1^B_*u*_	6.18	4.52	5.47	4.47	6.30	5.25	5.74	5.46	6.39	5.98	5.89	6.28	6.40	7.04	5.32	6.57	6.18
Hexatriene	2 ^1^A_*g*_	5.08		2.98	3.03	5.92		3.31	4.11	7.63		5.37	4.59	7.69		5.50	5.40	5.09
	1 ^1^B_*u*_	5.07	3.46	3.93	3.39	5.21	4.15	4.73	4.32	5.35	5.08	4.58	5.25	5.43	6.13	5.00	5.65	5.10
Octatetraene	2 ^1^A_*g*_	4.20		4.46	2.46	5.05		2.77	3.42	6.39		4.75	4.28	6.93		4.75	4.26	4.47
	1 ^1^B_*u*_	4.35	2.82	3.66	2.76	4.49	3.47	4.02	3.64	4.67	4.44	4.07	4.62	4.79	5.18	3.99	5.04	4.66
Acetone	1 ^1^A_2_	4.21	3.51	5.00	4.67	4.40	3.28	5.17	6.12	4.47	3.47	5.25	5.16	5.15	4.43	5.35	5.67	4.40
	2 ^1^A_1_	8.49		8.77	9.97	9.75		7.99	9.39	10.15		6.10	8.33	12.49		10.51	12.28	9.40
	1 ^1^B_1_	8.13		7.07	8.74	8.72		8.40	9.20	9.00		8.97	9.50	9.79		9.29	10.22	9.10
Formaldehyde	1 ^1^A_2_	3.80	3.08	4.63	4.20	3.92	2.83	4.71	4.58	3.94	3.04	4.70	4.60	4.51	1.42	4.76	5.04	3.88
	1 ^1^B_1_	8.82		9.41	8.84	9.03		9.58	9.64	9.12		9.61	9.70	9.65		9.55	10.03	9.10
	2 ^1^A_1_	10.47		7.55	10.16	10.60		7.67	11.51	9.88		8.43	12.20	9.63		6.17	8.02	9.30
Formamide	1 ^1^A^″^	5.45	4.92	6.48	6.16	5.62	4.33	6.63	6.49	5.65	4.45	6.64	6.52	6.49	5.20	6.71	7.03	5.63
	2 ^1^A^′^	8.54		5.14	5.91	8.67		5.47	6.06	7.96		4.43	6.69	12.32		6.81	6.32	7.39
	3 ^1^A^′^	10.64		8.38	9.92	11.80		6.55	7.71	12.17		8.71	14.42	13.87		8.21	10.92	
Adenine	2 ^1^A^′^	5.10		3.29	5.72	5.46		3.44	6.89	5.62		3.08	4.89	6.31		3.41	5.07	5.25
	3 ^1^A^′^	4.69		4.39	4.87	5.28		3.70	5.78	5.69		4.03	6.19	6.18		3.95	7.06	5.25
	4 ^1^A^′^	5.91		3.71	8.80	6.65		4.01	4.61	7.40		4.36	4.96	8.97		5.24	5.94	
	1 ^1^A^″^	4.28	4.05	4.79	4.79	5.10	4.18	5.13	5.62	5.53	5.39	5.15	6.22	7.04		5.76	7.85	5.12
	2 ^1^A^″^	5.02		5.24	5.38	5.73		5.71	6.19	6.14		5.59	6.78	7.50		5.21	6.78	5.75
Benzene	1 ^1^B_1*u*_	6.27		4.53	4.95	6.39		5.36	6.17	8.48		5.80	5.98	6.20		5.77	5.45	6.54
	1 ^1^B_2*u*_	5.29	5.57	3.13	5.13	5.51	6.24	5.30	6.28	6.61	6.72	3.90	6.19	6.05	7.76	5.68	5.31	5.08
	1 ^1^E_1*u*_	7.54		5.19	4.82	7.71		3.88	5.47	7.79		5.91	5.37	8.09		3.60	5.14	7.13
	1 ^1^E_2*g*_	8.32		6.77	5.30	9.20		6.91	7.73	9.93		8.64	9.72	10.62		7.85	9.01	8.41
Naphthalene	2 ^1^A_*g*_	5.85		4.99	9.87	6.32		3.59	5.69	6.63		5.30	5.53	7.27		5.79	5.92	5.87
	3 ^1^A_*g*_	6.24		4.35	9.36	7.11		4.18	4.09	8.70		5.26	4.18	9.40		6.65	4.80	6.67
	1 ^1^B_2*u*_	4.25		3.43	3.98	4.61		4.39	4.96	4.98		3.40	5.58	5.02		3.08	3.94	4.77
	2 ^1^B_2*u*_	6.12		4.70	6.09	6.49		4.34	7.18	6.86		5.63	7.85	7.20		5.39	4.65	6.33
	3 ^1^B_2*u*_	7.89		5.40	8.79	8.32		5.62	9.34	8.85		6.67	9.49	9.61		7.23	10.24	
	1 ^1^B_3*u*_	4.23	3.73	4.14	3.44	4.52	4.23	4.37	4.46	4.72	4.77	2.55	4.55	5.18	5.46	2.68	4.31	4.24
	2 ^1^B_3*u*_	6.23		2.82	3.11	6.49		2.89	4.20	6.64		4.87	4.38	7.04		5.44	4.15	6.06
	3 ^1^B_3*u*_	8.02		5.68	3.32	8.93		5.04	4.00	9.65		6.08	5.04	12.10		6.75	5.31	
	1 ^1^B_1*g*_	5.03		2.97	3.74	5.75		4.34	5.05	6.56		5.96	6.72	6.72		5.00	5.43	5.99
	2 ^1^B_1*g*_	6.49		4.57	2.98	6.69		3.70	4.03	6.98		5.88	7.08	7.87		4.50	4.85	6.47
MSD		–0.15		–1.16	–0.77	0.27		–1.06	–0.18	0.70		–0.68	0.29	1.23		–0.51	0.12	
MAD		0.39		1.32	1.40	0.35		1.28	0.94	0.70		0.94	0.79	1.26		0.94	0.92	
MSD [x-cDFT]^b^		–0.08	–0.98	–0.36	–0.67	0.13	–0.68	0.04	0.80	0.35	–0.11	–0.26	0.59	0.69	0.53	0.02	0.66	
MAD [x-cDFT]^b^		0.25	1.08	0.81	0.90	0.17	0.91	0.55	0.80	0.35	0.60	0.80	0.60	0.69	1.17	0.81	0.66	

a Theoretical Best Estimates (TBE) are taken from ref (98).

b Deviations for states that are accessible with x-cDFT.

The mean absolute deviations in Table 1 show that our new COOX method yields similar accuracies as x-cDFT or t-cDFT and a clear trend of improvement with the quaility of the chosen exchange-correlation functional (PBE < PBE0 < ωB97-X). To remove the computational overhead due to the LR-TDDFT calculation required for t-cDFT and COOX, we also show the results obtained with a sTDA reference in Table S1. While the average errors for t-cDFT increase, the errors for COOX remain virtually the same.

The results for a small benchmark set of triplet excitations is shown in Table 2. By default the scaled version of COOX has been employed apart from the 1^3^B_*u*_ state of octatetraene with ωB97-X/def2-TZVP due to convergence issues. Again, COOX shows a clear trend with improving quality of the exchange-correlation functional and yields at least similar and in most cases more accurate results than both x-cDFT and t-cDFT.

**Table 2:** Benchmark of Triplet Excitations Energies [eV] from TDA-TDDFT Reference^a^

		PBE/def2-TZVP				PBE0/def2-TZVP				ωB97-X/def2-TZVP				HF/def2-TZVP				
Molecule	State	TDA	x-cDFT	t-cDFT	COOX	TDA	x-cDFT	t-cDFT	COOX	TDA	x-cDFT	t-cDFT	COOX	TDA	x-cDFT	t-cDFT	COOX	TBE
Ethylene	1 ^3^B_1*u*_	4.50	4.59	4.28	4.45	4.30	4.40	4.00	4.24	4.44	4.56	4.15	4.41	3.58	3.69	6.18	4.29	4.50
Butadiene	1 ^3^A_*g*_	5.19		3.60	4.23	5.02		3.92	3.62	5.06		2.34	3.88	4.33		4.22	4.62	5.08
	1 ^3^B_*u*_	3.15	3.27	2.76	5.05	3.07	3.33	2.61	4.82	3.23	3.67	2.20	3.32	2.62	3.61	1.06	3.03	3.20
Hexatriene	1 ^3^A_*g*_	4.20		2.19	3.80	4.11		1.99	2.98	4.20		2.00	2.91	3.56		4.31	3.62	4.15
	1 ^3^B_*u*_	2.45	2.55	2.14	2.46	2.42	2.68	1.95	2.49	2.60	3.04	1.81	2.67	2.10	2.94	3.15	2.09	2.40
Octatetraene	1 ^3^A_*g*_	3.49		2.46	3.19	3.45		1.62	2.48	3.54		3.76	3.81	3.00		2.97	2.24	3.55
	1 ^3^B_*u*_	2.03	2.11	1.81	2.04	2.03	2.28	1.34	2.07	2.21	2.72	2.38	2.23^b^	1.78	2.87	0.72	2.24	2.20
Formaldehyde	1 ^3^A_2_	3.07	3.63	12.00	3.20	3.17	3.67	12.58	3.12	3.29	3.85	10.80	3.27	3.69	3.71	10.58	2.38	3.50
	1 ^3^A_1_	5.81		5.75	5.95	5.52		5.38	6.34	5.70		5.53	6.55	4.66		4.08	5.76	5.87
MSD		–0.06		0.28	0.29	–0.15		0.11	0.29	–0.02		0.06	–0.32	–0.57		0.31	–0.46	
MAD		0.11		1.61	0.37	0.16		1.91	0.42	0.08		1.65	0.57	0.61		1.84	0.47	
MSD [x-cDFT]^c^		–0.12	0.07	1.44	–0.02	–0.16	0.11	1.34	0.08	–0.01	0.41	1.11	0.02	–0.41	0.21	1.18	–0.35	
MAD [x-cDFT]^c^		0.14	0.10	1.96	0.13	0.17	0.15	2.29	0.32	0.10	0.41	1.88	0.15	0.48	0.53	2.63	0.37	

a Theoretical Best Estimates (TBE) are taken from ref. (98).

b regular COOX.

c deviations for states that are accessible with x-cDFT.

**Figure 1:**
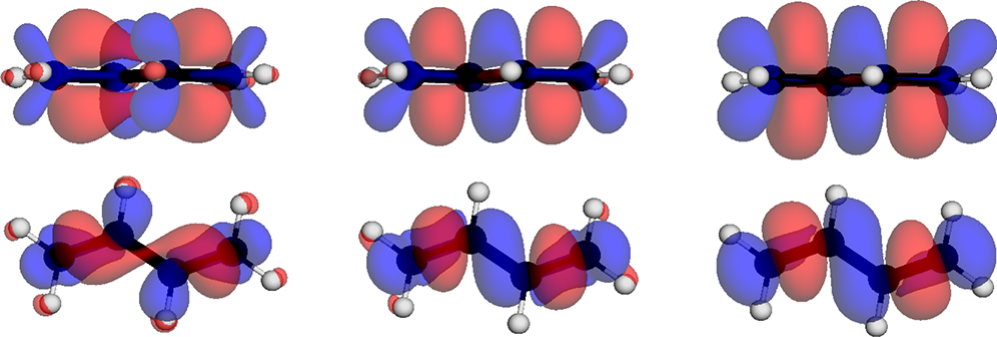
Difference densities of the 2A_*g*_ state of trans-butadiene obtained with LR-TDA-TDDFT@PBE/def2-TZVP (left), COOX@PBE/def2-TZVP (middle), and ADC(3)/def2-TZVP (right).

Focusing on the singlet excitations, it can be noted that the scaled version of COOX shows a strong improvement for degenerate excitations as in benzene (see also discussion below) and for those excitations with a strong double-excitation character like the 2A_*g*_ states of the small polyenes. It should be stressed that it can be assumed that the latter will probably not be described correctly by any of those methods, see, e.g., the discussion in ref (99). As it is also indicated by the approximate %T_1_/%T_2_ measures, COOX is in general—like other OO-TDDFT methods—capable of describing double-excitations. Nonetheless, the excited state density seems to be strongly improved as compared to the one obtained with LR-TDA-TDDFT as is shown in Figure 1. Here, the difference densities from LR-TDA-TDDFT, COOX, and ADC(3) are shown, where the latter method is capable of capturing the double-excitation character.^100^ The corresponding plots for 1B_*u*_ are shown in Figure S1.

Finally, it should be noted that the scaled version of COOX may overcorrect toward a single excitation character and therefore generating slightly lower excitation energies in some cases. These cases and possible improvements will be investigated in a future work.

### 3.2 Excited State Properties: Nuclear Derivatives

To analyze the quality and therefore usefulness of the excited state densities obtained with OO-TDDFT methods, the corresponding nuclear derivatives have been evaluated for a selection of molecules and compared to TDA-TDDFT results.

The nuclear derivatives are evaluated according to the formula for the ground-state derivatives according to the Hellmann–Feynman theorem^62,63^ including the Pulay-term.^64^ Following the notion in eq 12 that both  and  are stationary regarding ρ and λ_*c*_, the nuclear derivatives of the energy for the coordinate *x*_*A*_ of atom *A* is given as
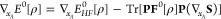
18Here, **P**/ρ represent the resulting one-electron density obtained with COOX,  is the Hellman-Feynman force of the unperturbed Hamiltonian (i.e., excluding the constraint potential **W**_*c*_), and  is the energy-weighted density matrix corresponding to . Note that this equation is also valid for the determination of nuclear derivatives for calculations employing fractional occupations^101^
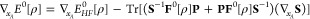
19

It should be stressed here that an exact agreement with LR-TDDFT is not expected since OO-TDDFT also includes (higher-order) nonlinear response of the electron density while the linear-response method misses significant parts of the electron–electron interactions.^41^ Thus, we focus our discussion on the comparison of excited state difference density plots and the corresponding nuclear derivatives, where the nuclear derivative vectors are given in atomic units and normalized to a maximum length of 1.0 Bohr.

**Figure 2:**
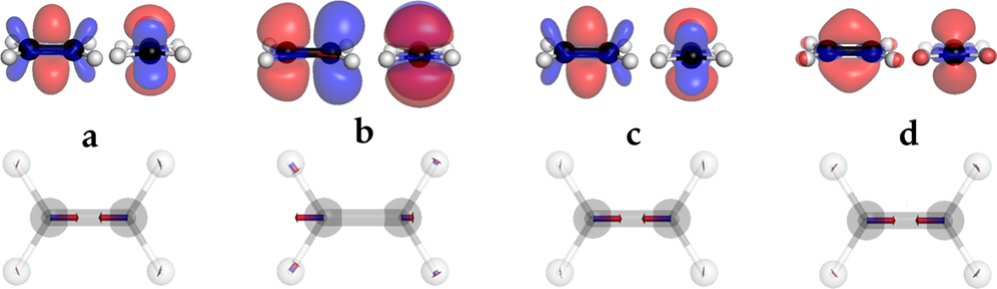
Singlet excited state (S_3_) difference densities (top) and nuclear derivatives (bottom) for ethylene obtained with *a)* x-cDFT, *b)* t-cDFT, *c)* COOX, and *d)* TDA-TDDFT (PBE/def2-TZVP). The nuclear derivative vectors are scaled by 0.5 for clarity.

In Figure 2, the results for the  state (S_3_) of ethylene is shown obtained with PBE/def2-TZVP for x-cDFT, t-cDFT, TDA-TDDFT, and the proposed COOX method. A first important point is that the x-cDFT method, that can only determine the lowest excited state, does not automatically deliver the S_1_ state of the employed method. Although in this case using PBE/def2-TZVP the S_3_ state is the first meaningful excitation, care must be taken to properly identify the excitation obtained with x-cDFT. As can be seen, the difference density of t-cDFT breaks the symmetry of the electronic structure and indicates a charge shift from one carbon center to another in contrast to x-cDFT, COOX, and TDA-TDDFT. This in turn also results in completely wrong nuclear derivatives for t-cDFT while those of x-cDFT and COOX resemble those of TDA-TDDFT. Further plots for acetamide and adenine can be found in Figures S2–S3 that also show that the COOX method provides qualitatively good densities and nuclear derivatives. In contrast, the x-cDFT method clearly fails to produce reasonable derivatives for adenine, indicating that the corresponding densities can be flawed which can also be concluded from the results for azobenzene obtained with post-SCF electron-correlation methods discussed below.

**Figure 3:**
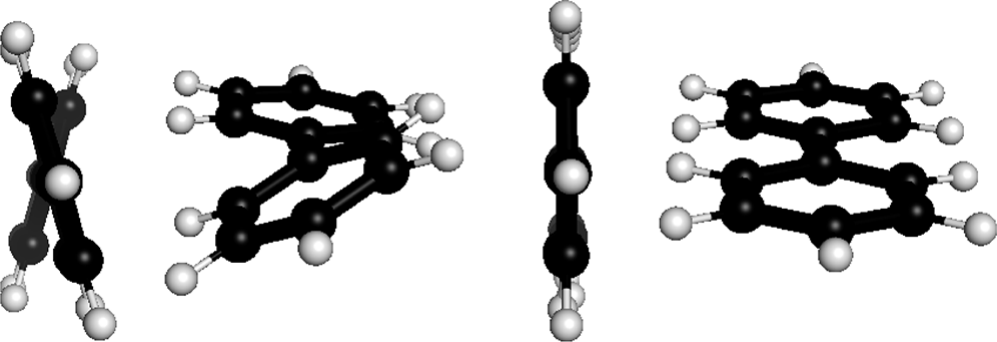
Optimized structures of biphenyl of S_0_ (left) and S_1_ (right) using excited state forces within the COOX scheme (PBE/def2-TZVP).

**Figure 4:**
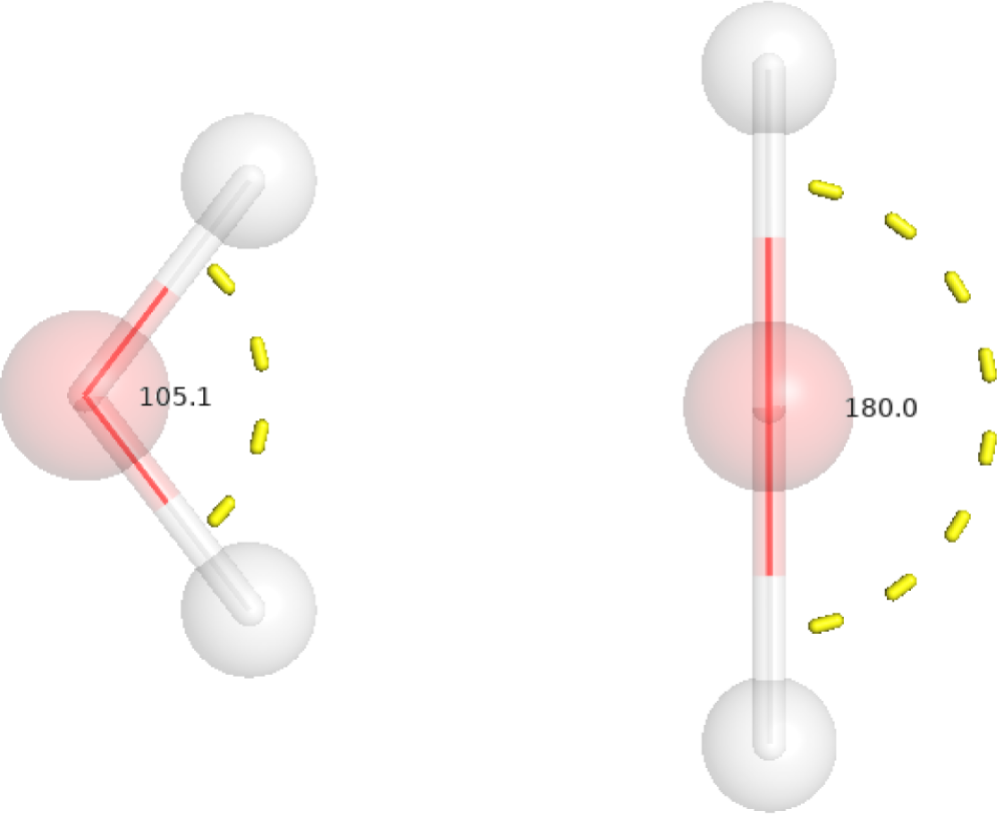
Optimized structure of water of S_0_ (left) and T_1_ (right) using excited state forces within the COOX scheme (PBE0/def2-TZVP).

We also employed the excited state forces obtained with COOX in the geometry optimization of the S_1_ state of the biphenyl molecule. While the two phenyl-rings are tilted toward each other in the ground-state structure, the S_1_ geometry is planar as can be seen in Figure 3. Compared to the S_1_ geometry obtained with TDA-TDDFT, the differences are quite small with at most 0.08 Å for bond lengths and 2.1^◦^ for angles. We also optimized the lowest triplet excited state of water using COOX as shown in Figure 4 using PBE0/def2-TZVP, where the first triplet excited state has a linear geometry. As for LR-TDDFT, our COOX method yields a charateristically linear geometry for the T_1_ state. The convergence behavior of the optimization process is similar to the one with LR-TDDFT, implying that COOX provides reliably accurate nuclear forces that can also be used in nonadiabatic molecular dynamics simulations.

As already indicated, the density resulting from t-cDFT is completely wrong and therefore unusable for subsequent excited state property calculations. Although x-cDFT densities seem to deliver good results in some cases like the S_3_ forces of ethylene, they fail to produce adequate results in other cases. In contrast, the COOX method yields in all test cases reasonable results similar to those obtained within a linear-response calculation.

### 3.3 Handling Degeneracy: Excited States of Benzene

As mentioned in the theory section, restricted singlet excitations with COOX can result in unwanted double-excitations due to degenerate HOMOs. We investigate this issue at the example of benzene, where the HOMO and the LUMO are degenerate.

As can be seen in Table 3, the COOX excitation energies obtained with the modified constraint potential in eq 14 are in good agreement with those of TDA-TDDFT, similar to the one result of x-cDFT for S_1_. However, for an unmodified restricted COOX calculation the S_1_/S_2_ excitation energy is 10.33 eV and further analysis shows that two electrons have been excited. Note that the x-cDFT result for S_1_ coincides with the result of an unrestricted COOX calculation (result obtained without spin purification).^58,59^

**Table 3:** Singlet Excitations [eV] of Benzene Using PBE/def2-TZVP

State	x-cDFT	t-cDFT	COOX	TDA-TDDFT
S_1_	5.14	5.51	5.32^a^	5.27
S_2_		6.39	4.95^a^	6.27
S_3_		7.04	7.51	7.01
S_4_		7.05	7.51	7.01

a scaled COOX calculation (eq 14).

**Figure 5:**
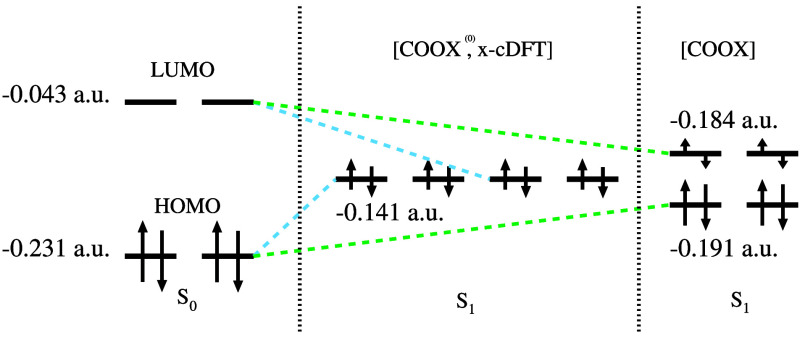
HOMO/LUMO orbital energies and occupancies for S_0_ and S_1_ of benzene at the PBE/def2-TZVP level of theory. The S_1_ state is calculated with both the unmodified and the scaled (eq 14) COOX methods, where the unmodified version delivers the same orbital energies as x-cDFT. The arrow lengths represent occupations of 1, 3/4, 1/2, and 1/4, respectively.

The reason for the failure of the standard COOX method can be easily seen from the HOMO/LUMO orbital energies depicted in Figure 5 (middle) using PBE/def2-TZVP. Here, the application of the unmodified constraint potential (COOX^(0)^) leads to a complete collapse of the energy gap resulting in four degenerate orbitals. Therefore, Fermi-smearing equally distributes half of an α and β electron into those orbitals, resulting in an unwanted double excitation. In contrast, within an unrestricted COOX calculation the constraint is only applied to the α spin channel, which preserves the HOMO–LUMO energy gap of the β orbitals and therefore a single-electron excitation is ensured. To obtain an accurate restricted wave function for S_1_, the scaled COOX constraint, as depicted on the right in Figure 5, has to be employed. This modified constraint delivers a significant HOMO–LUMO gap of 7 mHartree so that the two degenerate LUMOs are occupied by 0.25 α- and β-electrons each. The orbital energies for S_0_/S_1_ for several methods can be found in the Supporting Information.

**Figure 6:**
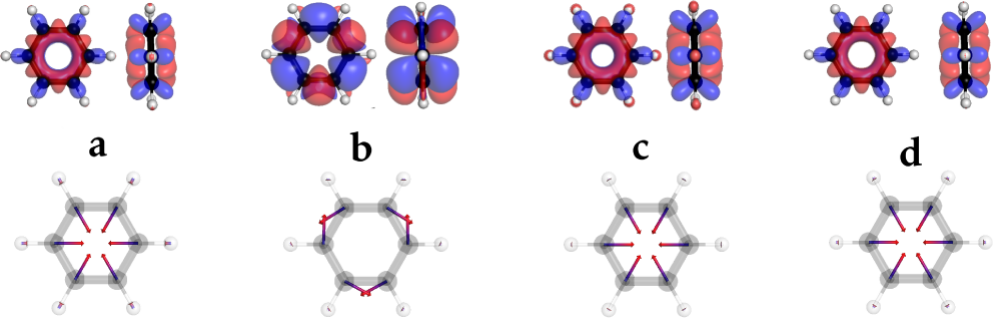
Excited state (S_1_) difference densities (top) and nuclear forces (bottom) of benzene obtained with *a)* x-cDFT, *b)* t-cDFT, *c)* COOX, and *d)* TDA-TDDFT (PBE/def2-TZVP).

Additionally, the excited state difference density and the corresponding nuclear forces for S_1_ of benzene is shown in Figure 6. Again, t-cDFT results show the wrong symmetry and completely wrong nuclear forces while x-cDFT and COOX results resemble those of linear-response TDA-TDDFT.

### 3.4 Intermolecular Charge-Transfer Excitations

A well-known issue of LR-TDDFT is the wrong description of intermolecular charge-transfer excitations. It has also been shown^33,35^ that OO-TDDFT methods strongly improve those results due to the capture of higher order, nonlinear terms.^41^ We evaluated the intermolecular charge-transfer energies for the systems [NH_3_···F_2_], [C_2_H_4_···C_2_F_4_], and [Benzene···TCNE]. The geometries as well as the corresponding best estimate were taken from refs. (102,103). The results for several functionals are given in Table 4.

**Table 4:** Charge Transfer Excitation Energies for Selected Systems in eV. A def2-TZVP Basis is used Throughout

Molecule	Best Est.	Method	x-cDFT	t-cDFT	COOX	TDA-TDDFT
NH_3_ ··· F_2_	9.46	PBE	3.47	9.78	7.23	0.65
		TPSS	3.66	10.10	7.51	1.02
		BR89BC95	3.69	9.89	7.28	0.47
		PBE0	5.75	11.87	10.10	3.49
		PW6B95	5.99	11.93	10.02	3.66
		PBEh-3c	7.46	13.69	12.27	5.34
		ωB97-X	8.63	13.46	13.43	6.93
C_2_H_4_ ··· C_2_F_4_	12.63	PBE	6.56	9.61	9.57	5.04
		TPSS	6.99	9.90	9.85	5.37
		BR89BC95	6.21	9.98	9.95	5.35
		PBE0	7.30	11.54	11.34	7.14
		PW6B95	7.12	11.69	11.47	7.37
		PBEh-3c	8.56	7.76	13.13	8.66
		ωB97-X	7.60	6.99	13.70	10.43
C_6_H_6_ ··· C_6_N_4_	3.59	PBE	2.65	3.66	3.71	1.35
		TPSS	2.73	3.69	3.75	1.40
		BR89BC95	2.74	3.72	3.79	1.46
		PBE0	3.68	4.03	4.16	2.08
		PW6B95	3.78	4.07	4.20	2.17
		PBEh-3c	3.98	4.42	4.61	2.62
		ωB97-X	3.83	4.48	4.94	3.58

The HOMO–LUMO singlet excitations have been calculated with different pure, hybrid, and range-corrected GGA- or meta-GGA-functionals. These functionals are sorted with respect to an increasing contribution of exact exchange, where ωB97-X contains less (0.15771) short-range exact exchange than PBEh-3c (0.42), but has 100% long-range exact exchange. As expected the LR-TDA-TDDFT results improve with the amount of exact exchange, while t-cDFT and COOX improve on the results of pure functionals like PBE. In contrast, x-cDFT (spin-purified) underestimates the excitations energies significantly.

Although the COOX results strongly improve on those obtained with TDA-TDDFT, it is notable that COOX shows a stronger dependence on the underlying density functional approximation than ΔSCF. In particular for the first two systems, the results obtained with pure functionals like PBE clearly underestimate the excitation energy, while ωB97-X with 100% long-range exact exchange yields results close to the best estimates. To investigate the functional dependence, we also evaluated the excitation energies for the [C_2_H_4_ ··· C_2_F_4_] system with respect to the intermolecular distance for the PBE, PBE0, and ωB97-X functionals (see Figures S4, S5, and S6, respectively) similar to refs. (18,22).

While still strongly improving on the TDA-TDDFT results, the results obtained with COOX employing the PBE or PBE0 functionals still deviate from the ideal Coulombic profile for larger distances, while ΔSCF and conventional cDFT show the correct asymptotic behavior. Only with ωB97-X can COOX produce the correct profile for larger distances, so that the excitation energies coincide with those of conventional cDFT.

Closer inspection of the HOMO/LUMO orbitals of the COOX excited state (Figures S7, S8, S9) show that for the functionals PBE and PBE0 only a partial charge transfer (0.5) is achieved in contrast to the ωB97-X result. All PBE orbitals are localized on one of the molecules only and exhibit energetically degenerate HOMO and LUMO orbitals. Thus, the LUMO and LUMO+1 orbitals feature significant occupation numbers due to applied Fermi-smearing at 1000 K, resulting in a backflow of electron charges to the C_2_F_4_ molecule. In contrast, the orbitals obtained with ωB97-X feature a significant HOMO–LUMO-gap and therefore no spill of electronic charges into the LUMO. The charge transfer is realized by an even delocalization of the doubly occupied HOMO–1 orbital.

It is also interesting to note that the ΔSCF results are, as expected, hardly dependent on the type of functional, while the conventional cDFT shows a stronger dependence (similar to COOX), but both ΔSCF and cDFT show the correct asymptotic behavior for all functionals. However, the results for ωB97-X of both cDFT and COOX are closer to the best estimate for the intermolecular distance of 8.0 Å (12.63 eV from ref (104)) than ΔSCF. Finally, it should be mentioned that it is difficult to analyze the impact of the different types of density functional approximations due to the variational nature of the COOX method as they may converge toward distinct variational minima. Therefore, while warranted, a more in-depth analysis of long-range CT states is beyond the scope of this work.

### 3.5 Conical Intersections

Finally, we investigate the description of the S_0_-S_1_ conical intersection in the rotational potential energy profile of azobenzene. Here, it should be stressed that an overall accurate description of conical intersections will of course require the correct description of the multireference character of the electronic structure, which is not accounted for by any of the methods in our investigation, in which we primarily focus on the correct S0/S1 state ordering. We use the same structures as in ref (105) and evaluate the energies for PBE, PBE0, and Hartree–Fock (HF), where for the latter two calculations additional post-SCF electron-correlation energies have been evaluated using direct RPA^66−70^ and σ-RPA.^106−108^

**Figure 7:**
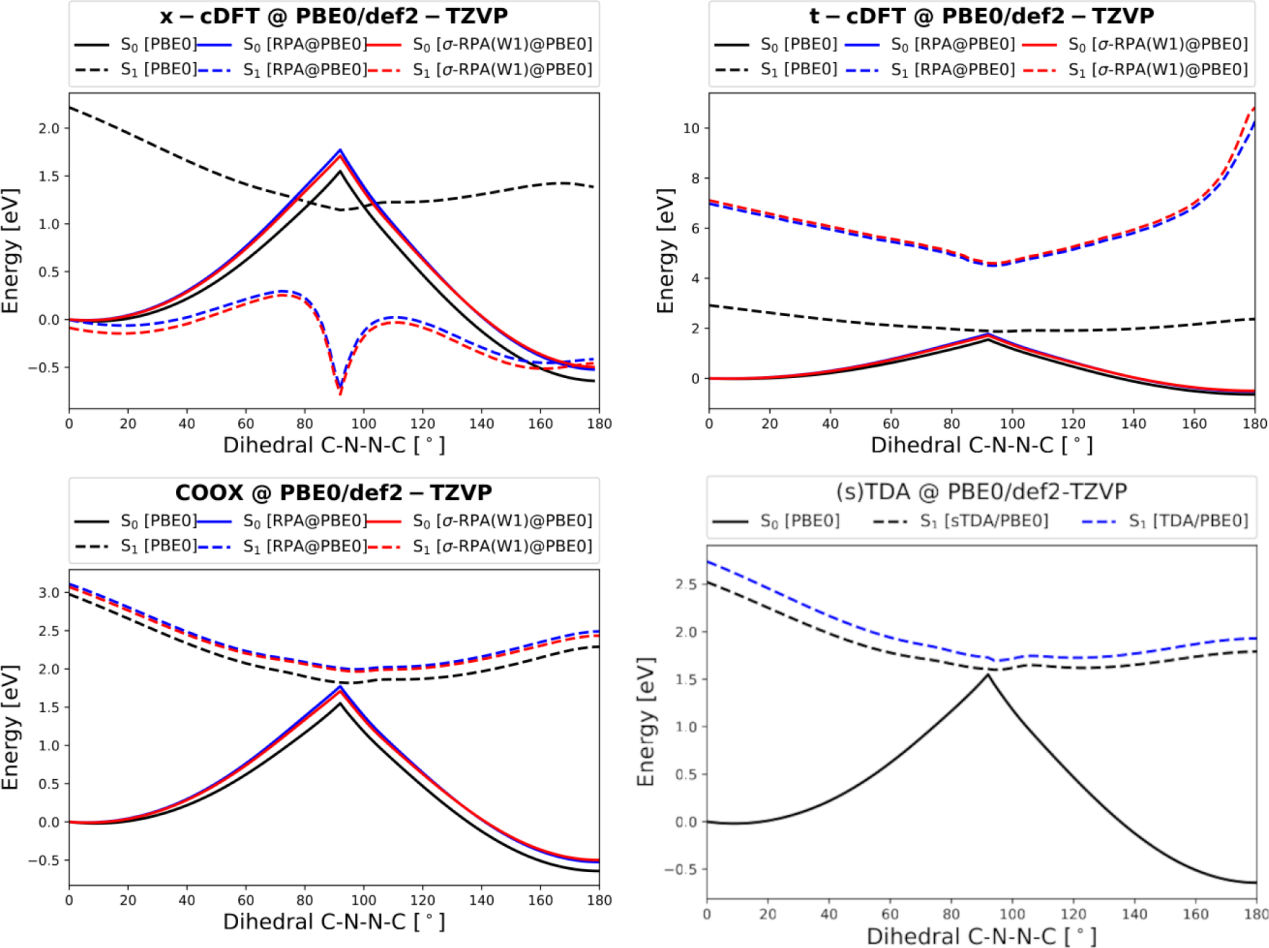
S_0_ and S_1_ for the rotational profile of the azobenzene isomerization at PBE0/def2-TZVP level of theory for x-cDFT, t-cDFT, COOX, and (simplified) TDA. Furthermore, the energy profile for the post-Kohn–Sham methods direct RPA and σ-RPA(W1) are shown.

In Figure 7 the plots for S_0_/S_1_ of azobenzene at the PBE0/def2-TZVP level of theory are shown. Compared to the reference values obtained with TDA and sTDA, respectively, x-cDFT yields overall too small excitation energies and also fails to obtain the correct state ordering around 90^◦^. In contrast, t-cDFT and COOX show a correct state ordering and also a improve on the overall quality. For example, the excitation energy at 0^◦^ is close to 3 eV similar to the result obtained with CASPT2/def2-TZVP.^105^

As a further analysis of the quality of the excited state electronic structure obtained with the different OO-TDDFT methods, the energies obtained with direct RPA and σ-RPA are shown as well. For both x-cDFT and t-cDFT, the results strongly deviate and deliver unreasonable excitation energies. In contrast, the correlated energies obtained with our COOX method provide a qualitatively correct picture again indicating the quality of the resulting excited state density.

The corresponding plots for PBE and HF can be found in Figures S10, S11. For PBE, the ground-state could not be converged at the conical intersection so that Fermi-smearing had to be applied for the SCF as well. For the HF calculations, the x-cDFT excited state calculations did not converge. As for PBE0, the COOX method delivers accurate results over the whole spectrum while x-cDFT and t-cDFT either fail to converge at the conical intersection or produce wrong results when direct RPA is applied.

## 4 Conclusion

We presented a new orbital-optimized excited state method by applying a constraint derived from the static parts of the excited state difference density. In contrast to the previously proposed x-cDFT and t-cDFT methods, our COOX method delivers both accurate excited state energies compared to similar methods and densities, which allows to further apply post-SCF electron correlation methods and the evaluation of excited state properties.

In comparison to ΔSCF-type methods, COOX can target specific excited states and does provide a stable optimization, i.e., it does not suffer from a collapse to the ground-state. Additionally, the resulting excited state wave functions conserve the Aufbau principle which allows the unmodified application of post-SCF methods (e.g., Laplace-transformation). Similar to t-cDFT, one potential additional bottleneck of our method is the need for a preceding LR-TDDFT calculation. We have shown that the constraints can also be formed from the results of simplified TDA calculations, thus eliminating virtually any additional computational cost as compared to x-cDFT or ΔSCF-type methods.

Based on these encouraging results, further investigations regarding the applicability of the presented COOX method will be subject to future works. For example, other excited state properties than nuclear forces and also its use in nonadiabatic molecular dynamics (NAMD) simulations will be investigated.

## Supporting Information Available

Derivation of COOX scaling factor from full LR-TDDFT, constraint expressions for fractional-occupation ground states, additional excited-state difference densities and gradients, additional benchmark data, analysis of asymptotic behavior for CT states, and azobenzene isomerization profile for PBE and HF (PDF)

Orbital energies for the benzene S_0_–S_1_ transition for regular and scaled COOX (ZIP)
