# Supplementary material for: A Constraint-Based Orbital-Optimized Excited State Method (COOX)
Source: J Chem Theory Comput. 2024 Sep 30;20(19):8461–73. doi: 10.1021/acs.jctc.4c00467 (PMC11465468; doi:10.1021/acs.jctc.4c00467)
Supplement: Supplementary file 1 — ct4c00467_si_001.pdf [file ct4c00467_si_001.pdf]

Supporting Information: A Constraint-Based Orbital-Optimized Excited State Method (COOX)

Jörg Kussmann,* Yannick Lemke, Anthea Weinbrenner, and Christian Ochsenfeld*

Chair of Theoretical Chemistry, Department of Chemistry, Ludwig-Maximilians-Universität in Munich (LMU), Butenandtstr. 5, D-81377 München, Germany

E-mail: joerg.kussmann@uni-muenchen.de; christian.ochsenfeld@uni-muenchen.de

LR-Based Constraint Modification for TDDFT/RPA

$$
\begin{aligned}
\tilde{\mathbf{P}}^0 &= \mathbf{S}^{\frac{1}{2}}\mathbf{P}^0\mathbf{S}^{\frac{1}{2}}, \quad \tilde{\mathbf{W}}_c = \mathbf{S}^{-\frac{1}{2}}\mathbf{W}_c\mathbf{S}^{-\frac{1}{2}} \\
\tilde{\mathbf{V}} &= \mathbf{S}^{\frac{1}{2}}\mathbf{\Delta P}^{\mathrm{virt}}\mathbf{S}^{\frac{1}{2}} = \mathbf{S}^{\frac{1}{2}}\mathbf{C}\left(\mathbf{X}\mathbf{X}^\dagger + \mathbf{Y}\mathbf{Y}^\dagger\right)\mathbf{C}^\dagger\mathbf{S}^{\frac{1}{2}} \\
\tilde{\mathbf{O}} &= \mathbf{S}^{\frac{1}{2}}\mathbf{\Delta P}^{\mathrm{occ}}\mathbf{S}^{\frac{1}{2}} = \mathbf{S}^{\frac{1}{2}}\mathbf{C}\left(\mathbf{X}^\dagger\mathbf{X} + \mathbf{Y}^\dagger\mathbf{Y}\right)\mathbf{C}^\dagger\mathbf{S}^{\frac{1}{2}} \\
\tilde{\mathbf{Z}} &= \mathbf{S}^{\frac{1}{2}}\mathbf{\Delta P}^{\mathrm{ov}}\mathbf{S}^{\frac{1}{2}} \\
\tilde{\mathbf{P}}^1_{\mathrm{LR}} &= \tilde{\mathbf{P}}^0 + \tilde{\mathbf{V}} - \tilde{\mathbf{O}} + \tilde{\mathbf{Z}} + \tilde{\mathbf{Z}}^\dagger
\end{aligned}
$$

with the (unperturbed) ground-state density $\mathbf{P}^0$ and the occupied-virtual part $\mathbf{Z}$ of the

excited state density $\mathbf{P}^1$.

$$\begin{aligned}
\mathrm{Tr}\left[\tilde{\mathbf{P}}^1_{\mathrm{LR}}\tilde{\mathbf{W}}_c\right] &= \mathrm{Tr}\left[\tilde{\mathbf{P}}^1_{\mathrm{LR}}\left(\tilde{\mathbf{V}}-\tilde{\mathbf{O}}\right)\right] \\
&= \mathrm{Tr}\left[\left(\tilde{\mathbf{P}}^0+\tilde{\mathbf{V}}-\tilde{\mathbf{O}}+\tilde{\mathbf{Z}}+\tilde{\mathbf{Z}}^\dagger\right)\left(\tilde{\mathbf{V}}-\tilde{\mathbf{O}}\right)\right] \\
&= -\mathrm{Tr}\left[\tilde{\mathbf{P}}^0\tilde{\mathbf{O}}\right]+\mathrm{Tr}\left[\tilde{\mathbf{V}}\tilde{\mathbf{V}}\right]+\mathrm{Tr}\left[\tilde{\mathbf{O}}\tilde{\mathbf{O}}\right] \\
&= \|\tilde{\mathbf{V}}\|^2+\|\tilde{\mathbf{O}}\|^2-\|\mathbf{X}\|^2-\|\mathbf{Y}\|^2 \\
&= 2\|\mathbf{X}\mathbf{X}^\dagger\|^2+2\|\mathbf{Y}\mathbf{Y}^\dagger\|^2+2\|\left(\mathbf{X}\mathbf{X}^\dagger\right)\left(\mathbf{Y}\mathbf{Y}^\dagger\right)\|^2+2\|\left(\mathbf{X}^\dagger\mathbf{X}\right)\left(\mathbf{Y}^\dagger\mathbf{Y}\right)\|^2 \\
&\quad -\|\mathbf{X}\|^2-\|\mathbf{Y}\|^2 \qquad (1)
\end{aligned}$$

LR-Based Constraint Modification for Fractional Occupations

As there are orbitals with fractional occupations, the strict assignment to occupied and virtual sub-spaces fails. Thus, the dimension of the transition coefficient matrix $\mathbf{X}_{vo}$ is $\mathrm{N}_{\mathrm{orb}}\times\mathrm{N}_{\mathrm{orb}}$, but the rows are scaled by $\sqrt{1-n_a}$ and the columns by $\sqrt{n_i}$, respectively. Note that for ground-state containing fractional occupations it is not possible to determine a level-shifting factor f_v since $\mathrm{Tr}\left[\tilde{\mathbf{Z}}\left(\tilde{\mathbf{V}}-\tilde{\mathbf{O}}\right)\right]\neq 0$. Thus, a non-zero constant parameter has to be used:

$$N_c = -\mathrm{Tr}\left[\tilde{\mathbf{P}}^1_{\mathrm{LR}}\tilde{\mathbf{W}}_c\right] \qquad (2)$$

Orbital Energies for S_0/S_1 of Benzene

The svg-files can be opened in a regular browser. The cyan dots can be clicked to get further information with respect to the orbital mixing due to the transition from S_0 to S_1.

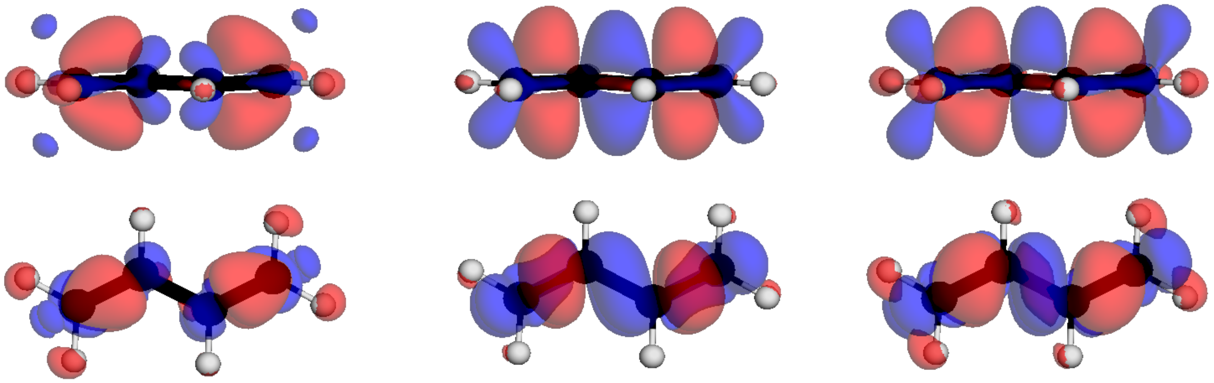

Figure S1: Difference densities of the $1B_u$ state of trans-butadiene obtanied with LR-TDA-TDDFT@PBE/def2-TZVP (left), COOX@PBE/def2-TZVP (middle), and ADC(3)/def2-TZVP (right).

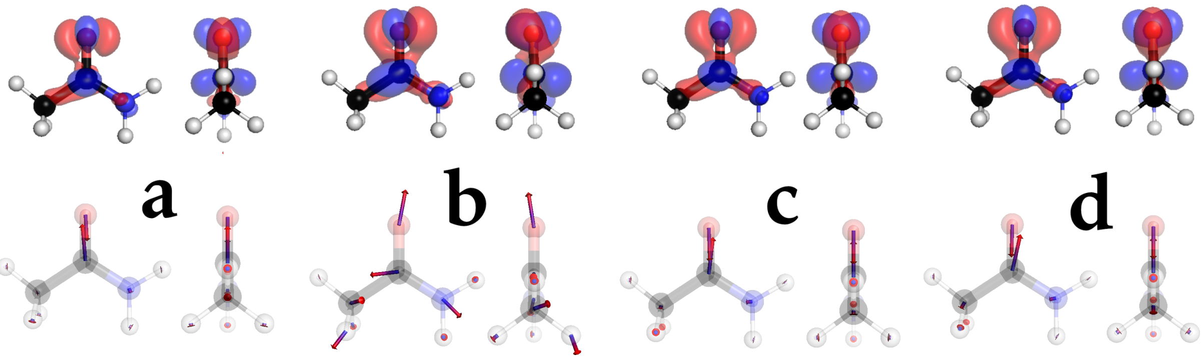

Figure S2: Excited state (S_1) difference density (top) and nuclear derivatives (bottom) of acetamide obtained with **a)** x-cDFT, **b)** t-cDFT, **c)** COOX, and **d)** TDA-TDDFT (PBE/def2-TZVP).

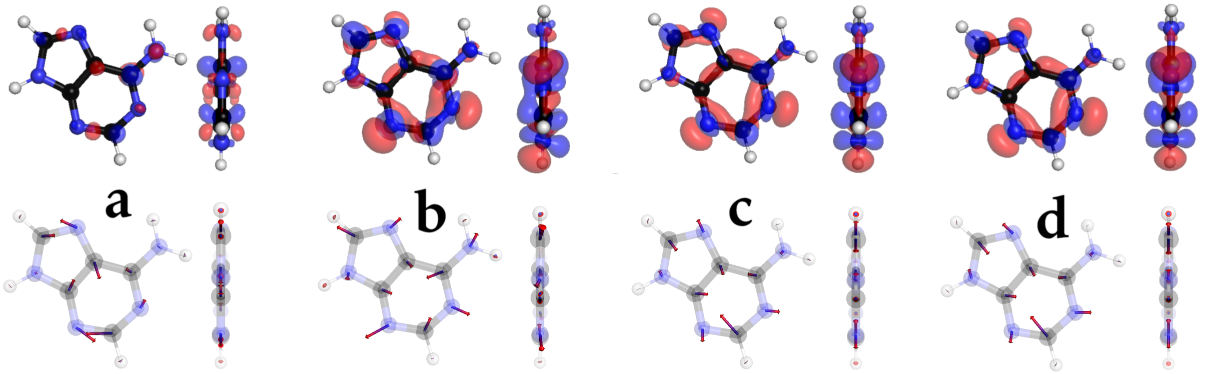

Figure S3: Excited state (S_1) difference density (top) and nuclear derivatives (bottom) of adenine obtained with **a)** x-cDFT, **b)** t-cDFT, **c)** COOX, and **d)** TDA-TDDFT (PBE/def2-TZVP).

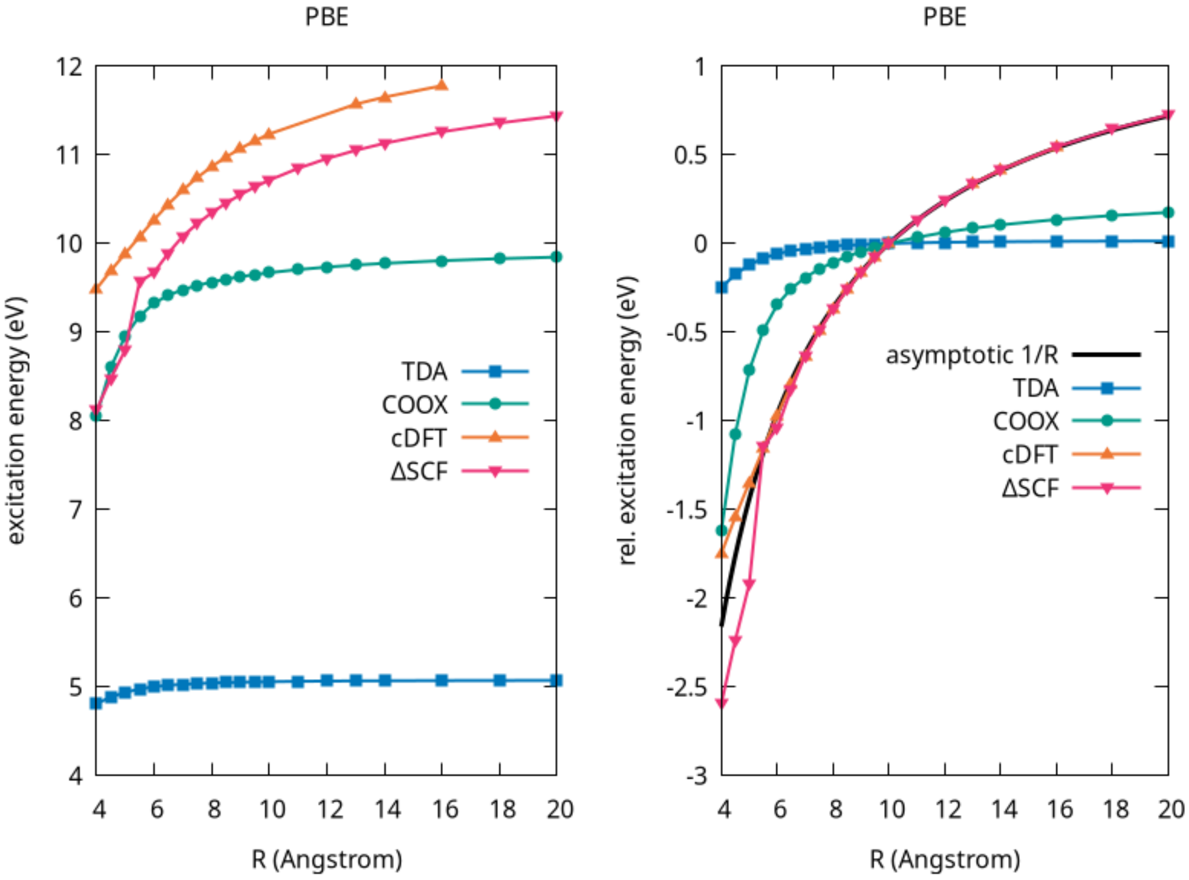

Figure S4: Excitation energy vs distance plot of [C_2H_4...C_2F_4] for lowest charge transfer state obtained with COOX at PBE/def2-TZVP level of theory. In the plot on the right the energies are shifted with respected to the respective value for a distance of 10Å to illustrate the asymptotic $1/r$ behavior.

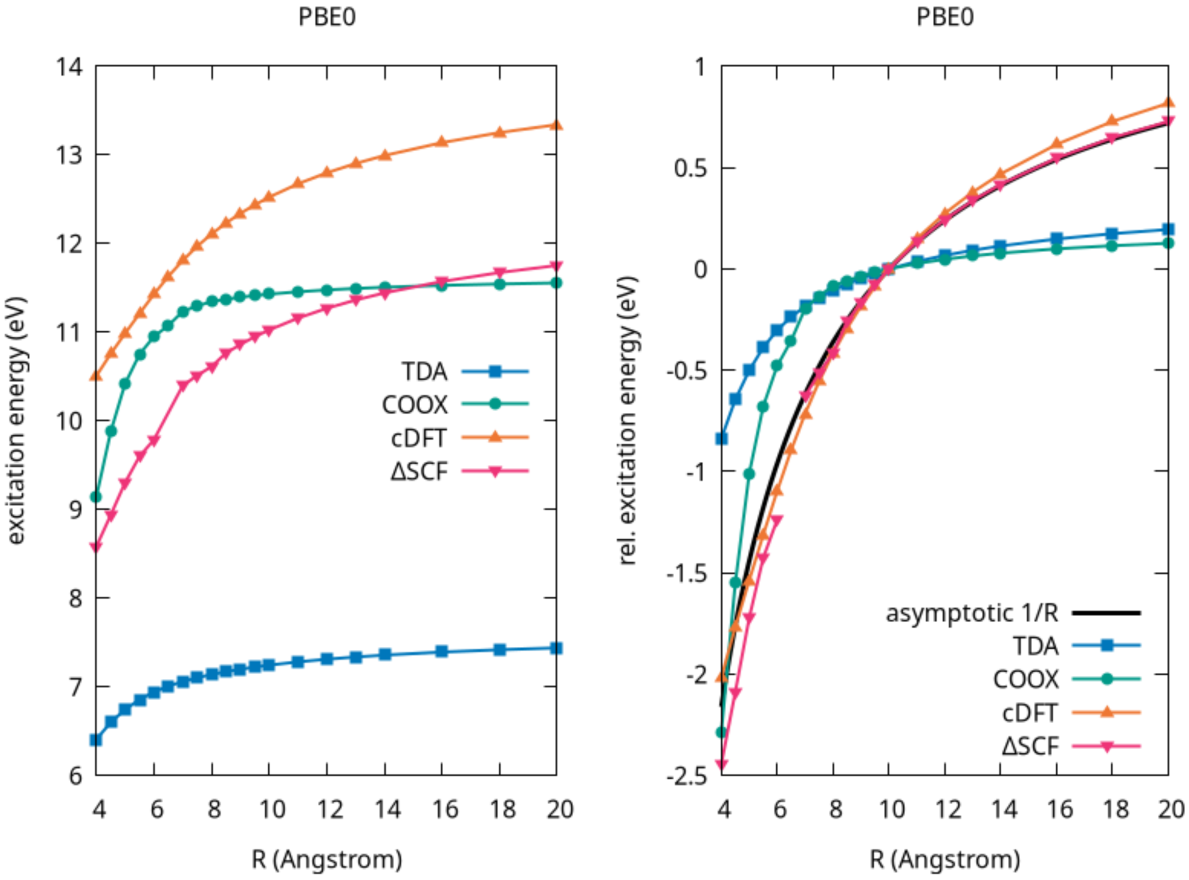

Figure S5: Excitation energy vs distance plot of [C_2H_4...C_2F_4] for lowest charge transfer state obtained with COOX at PBE0/def2-TZVP level of theory. In the plot on the right the energies are shifted with respected to the respective value for a distance of 10Å to illustrate the asymptotic $1/r$ behavior.

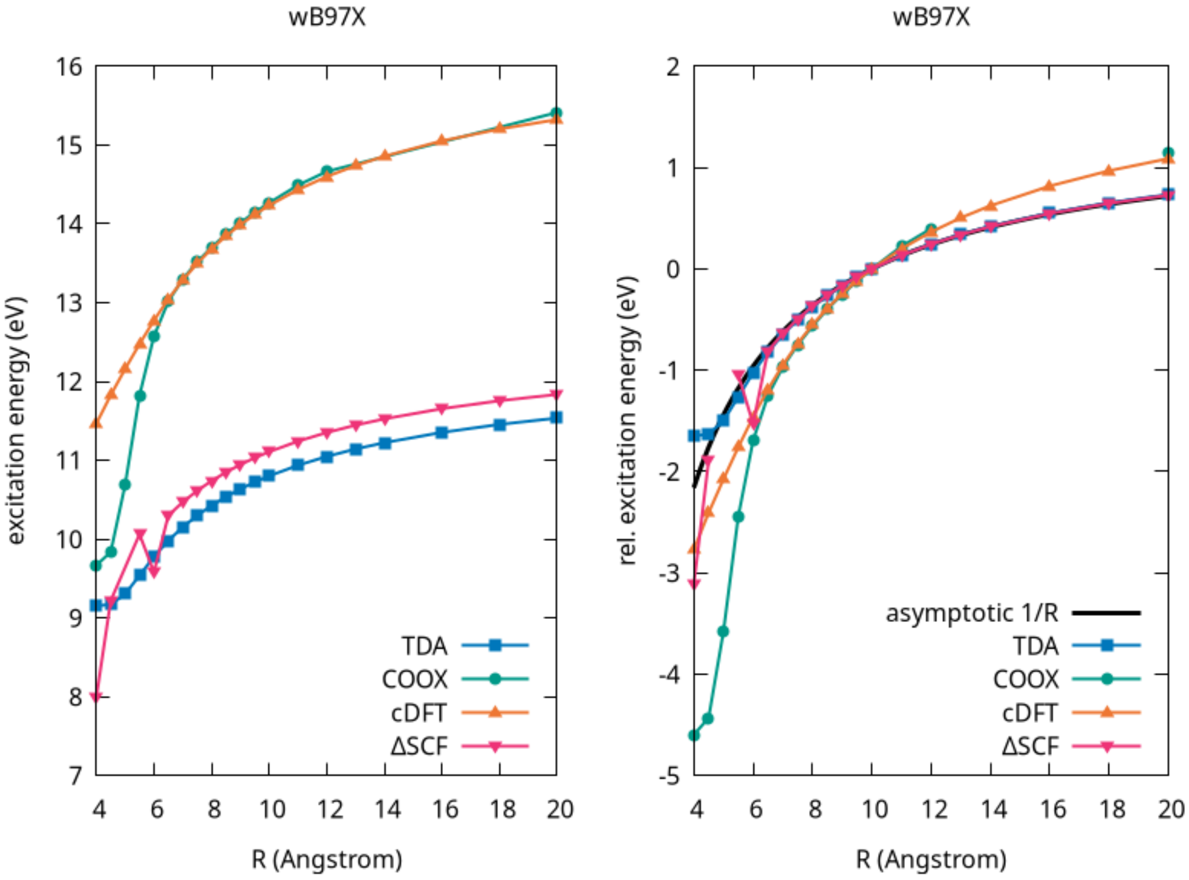

Figure S6: Excitation energy vs distance plot of [C_2H_4...C_2F_4] for lowest charge transfer state obtained with COOX at ωB97-X/def2-TZVP level of theory. In the plot on the right the energies are shifted with respected to the respective value for a distance of 10Å to illustrate the asymptotic $1/r$ behavior.

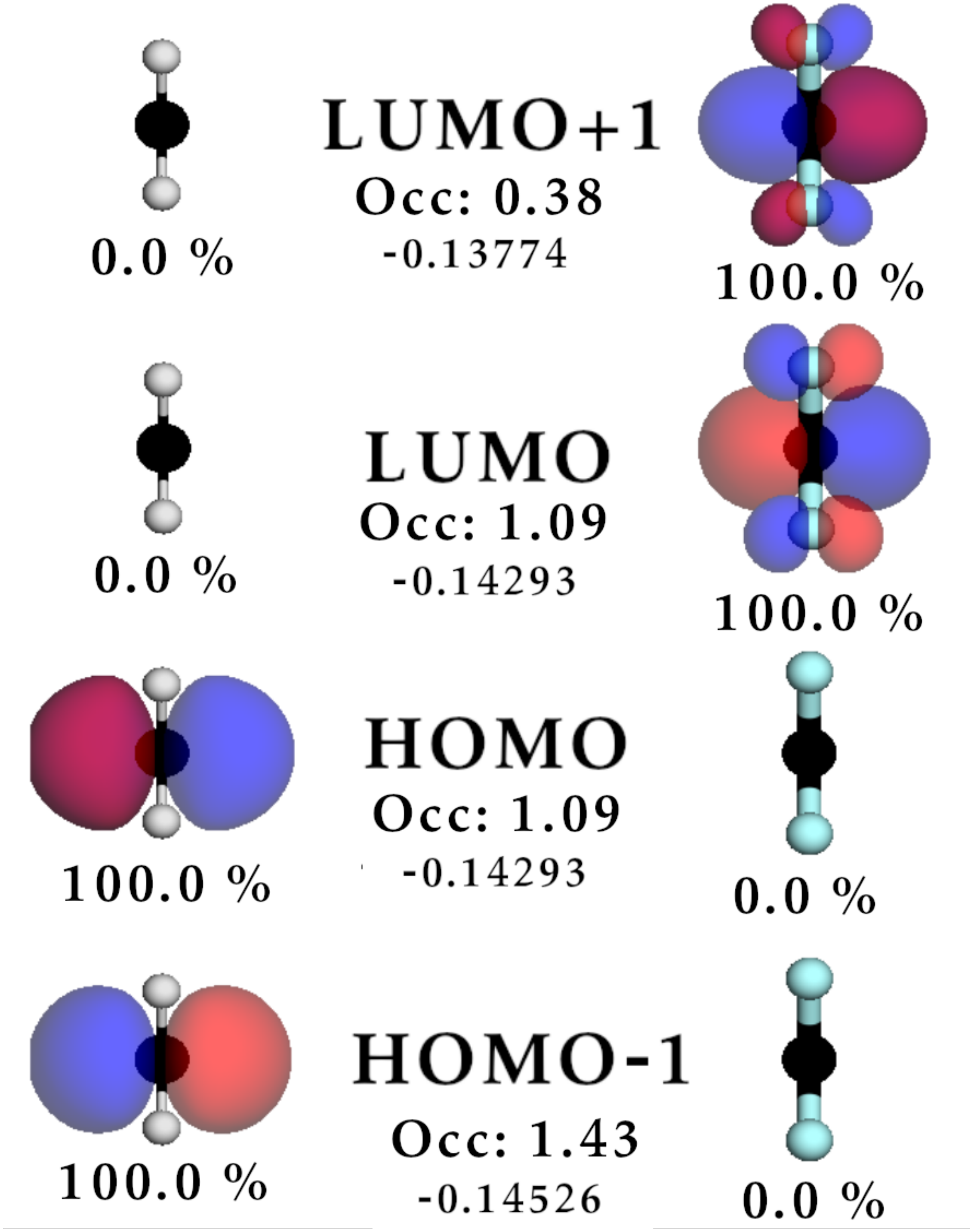

Figure S7: HOMO(-1)/LUMO(+1) orbitals of [C_2H_4...C_2F_4] for lowest charge transfer state obtained with COOX at PBE/def2-TZVP level of theory. The occupancy and energy of orbitals is shown as well as the localization in percent on the individual molecules. The latter have been obtained by Mulliken population analysis for the individual orbitals. The number of electrons transfered is 0.53 (Mulliken population analysis).

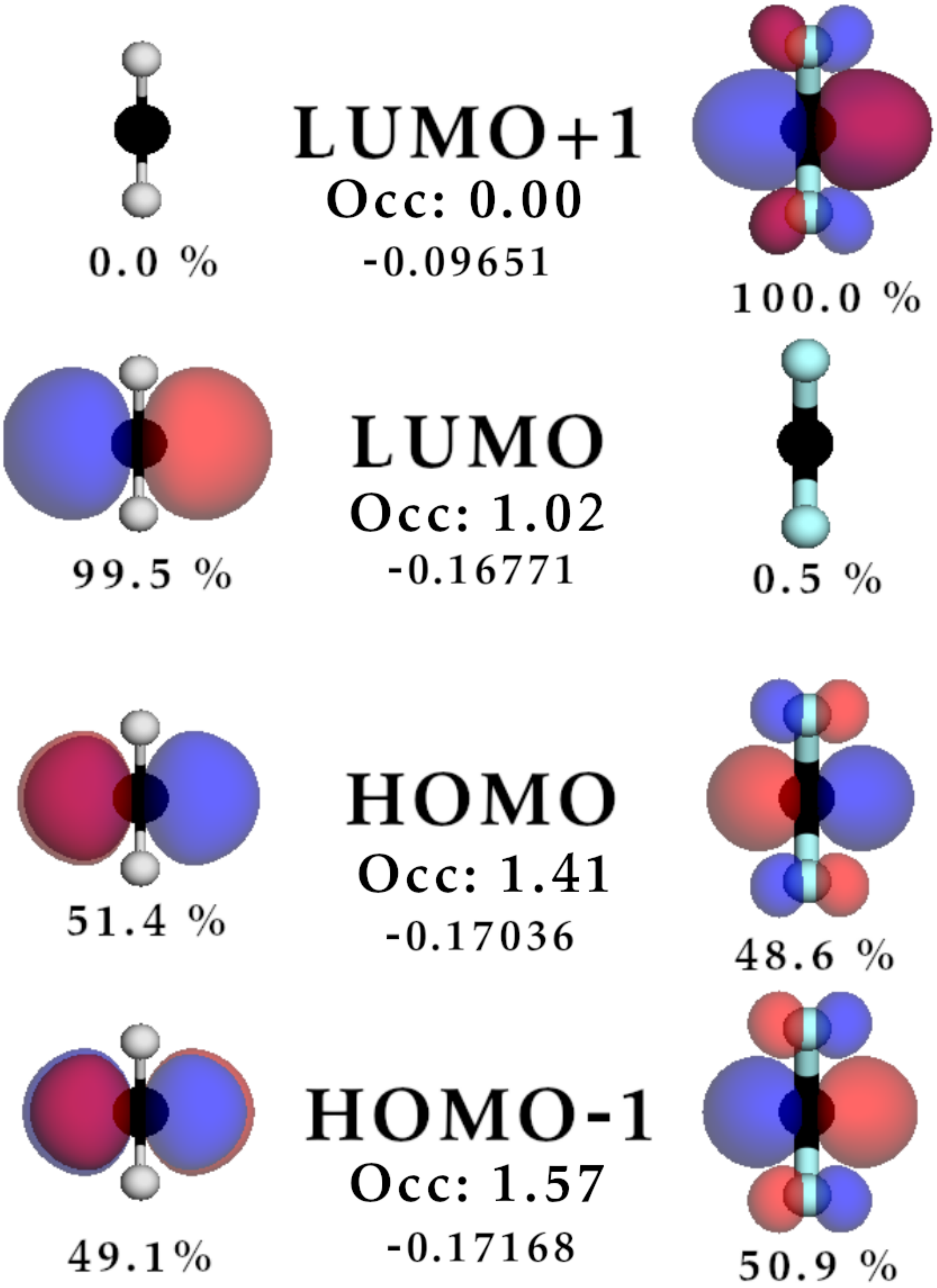

Figure S8: HOMO(-1)/LUMO(+1) orbitals of [C_2H_4...C_2F_4] for lowest charge transfer state obtained with COOX at PBE0/def2-TZVP level of theory. The occupancy and energy of orbitals is shown as well as the localization in percent on the individual molecules. The latter have been obtained by Mulliken population analysis for the individual orbitals. The number of electrons transfered is 0.51 (Mulliken population analysis).

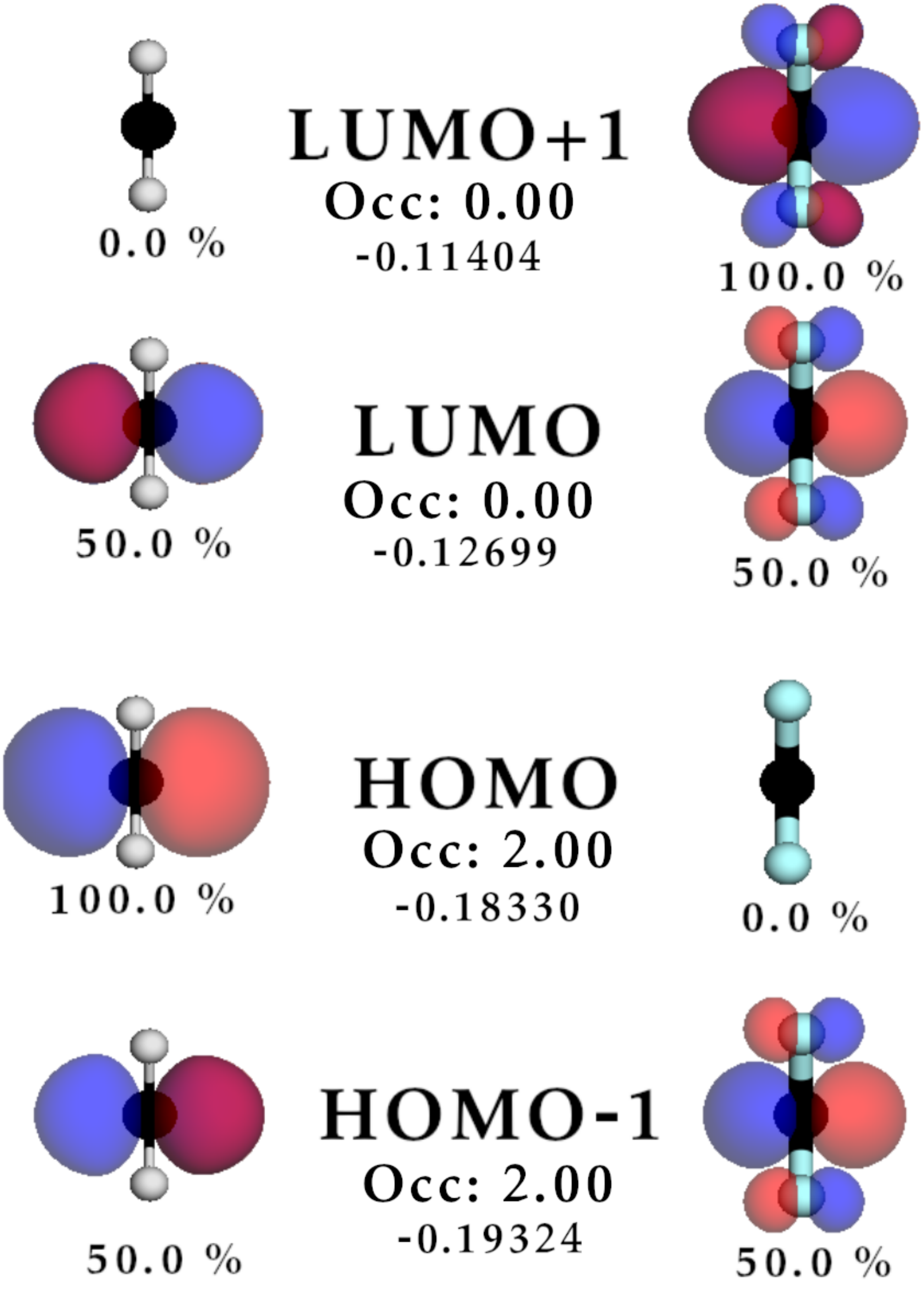

Figure S9: HOMO(-1)/LUMO(+1) orbitals of [C_2H_4...C_2F_4] for lowest charge transfer state obtained with COOX at ωB97-X/def2-TZVP level of theory. The occupancy and energy of orbitals is shown as well as the localization in percent on the individual molecules. The latter have been obtained by Mulliken population analysis for the individual orbitals. The number of electrons transfered is 0.98 (Mulliken population analysis).

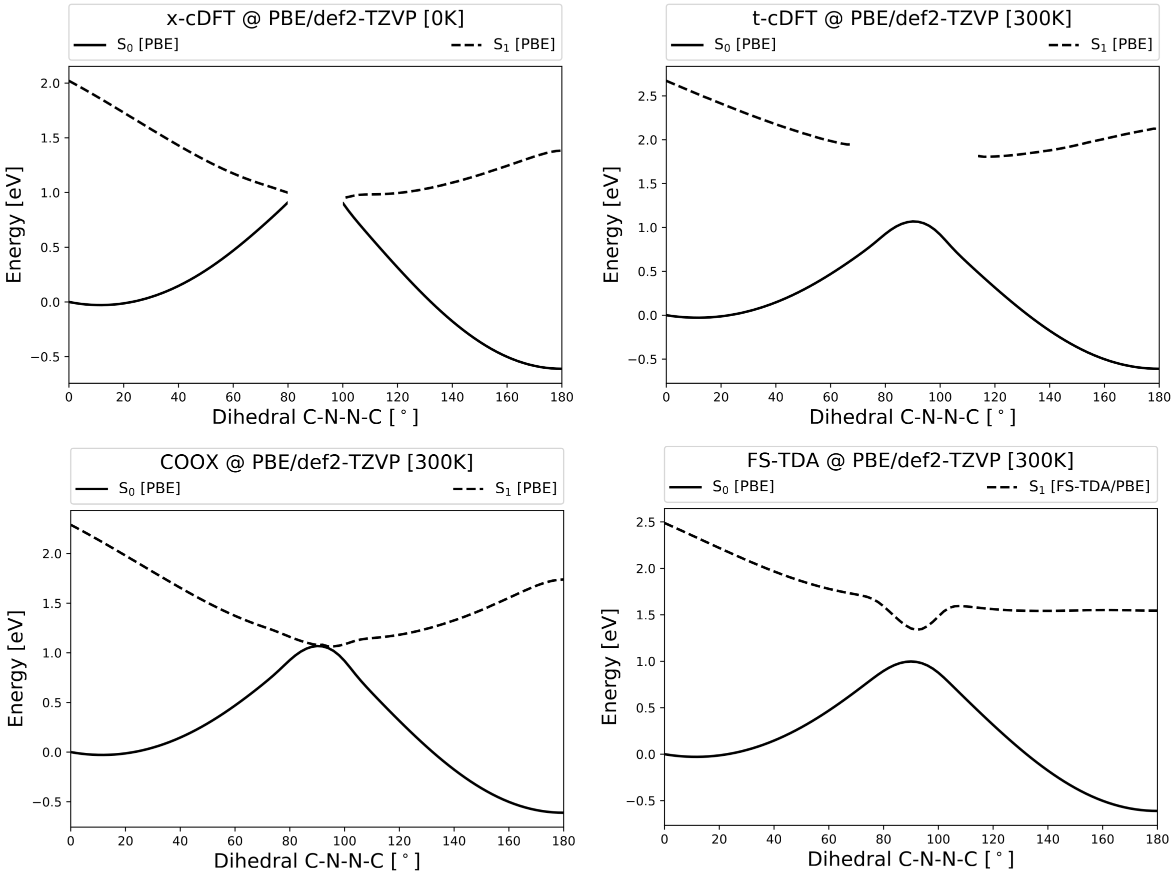

Figure S10: S_0 and S_1 for the rotational profile of the azobenzene isomerization at PBE/def2-TZVP level of theory for x-cDFT, t-cDFT, COOX, and simplified TDA. Note that Fermi-smearing had to be employed for the ground-state in order to converge the SCF calculation. Note that the constraint for x-cDFT only works for 0K, i.e., for an idempotent density operator. Furthermore, the t-cDFT calculations around 90° did not converge.

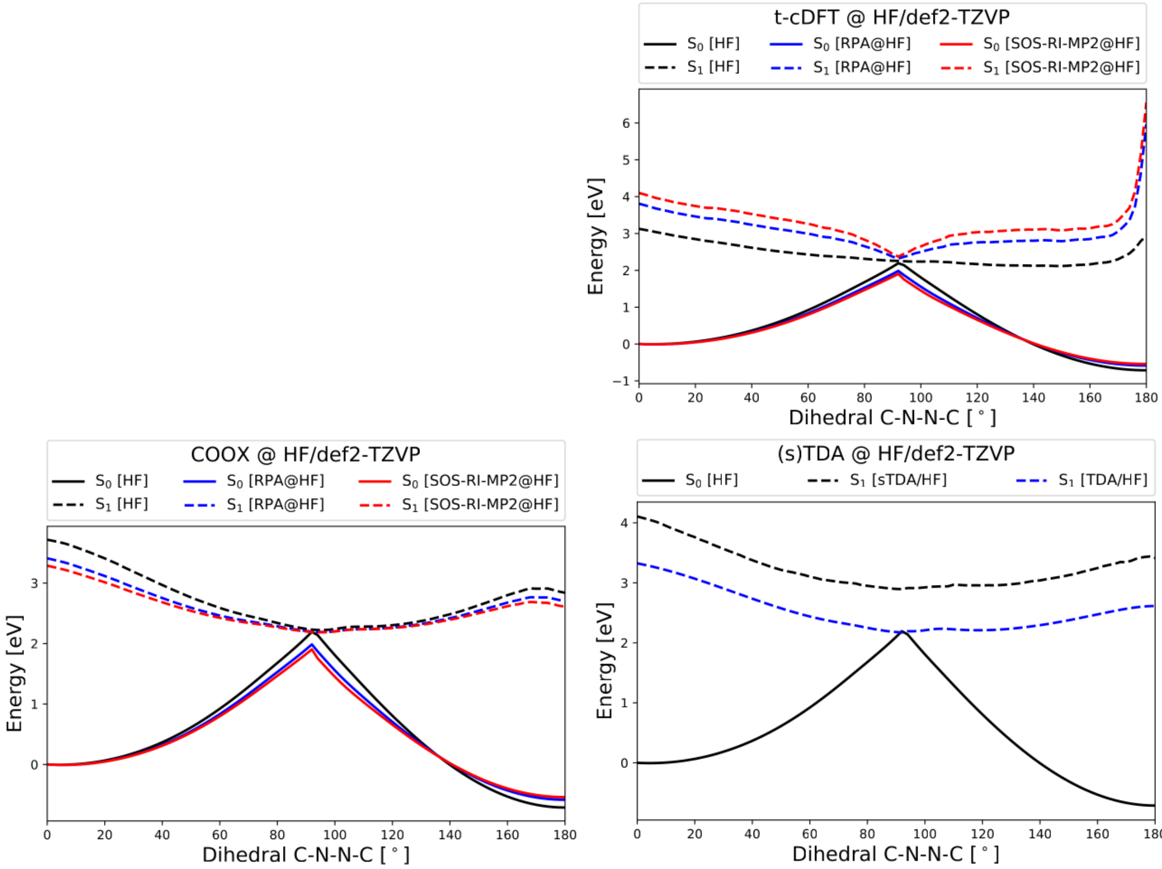

Figure S11: S_0 and S_1 for the rotational profile of the azobenzene isomerization at HF/def2-TZVP level of theory for t-cDFT, COOX, and simplified TDA. Furthermore, the energy profile for the post-SCF methods direct RPA and SOS-RI-MP2 are shown. The x-cDFT calculations for HF did not converge.

Table S1: Benchmark of singlet excitations energies [eV] from sTDA-reference.

Molecule	State	PBE/def2-TZVP			PBE0/def2-TZVP			ωB97-X/def2-TZVP			HF/def2-TZVP			
		TDA	t-cDFT	COOX	TDA	t-cDFT	COOX	TDA	t-cDFT	COOX	TDA	t-cDFT	COOX	TBE
Ethylene	1 $^1B_{1u}$	8.18	7.28	6.07	8.03	7.24	7.25	15.17	7.05	8.33	6.65	7.30	7.93	7.80
Butadiene	2 ^1A_g	6.24	3.71	4.03	6.91	4.05	5.55	12.94	5.26	7.27	7.25	5.05	7.29	6.55
	1 ^1B_u	6.03	4.83	4.22	6.14	5.32	5.27	12.10	4.77	6.22	5.88	6.16	6.64	6.18
Hexatriene	2 ^1A_g	5.03	3.04	2.97	5.74	3.50	4.02	11.40	4.62	5.00	6.98	5.58	7.71	5.09
	1 ^1B_u	4.95	3.94	3.21	5.02	4.47	4.21	10.38	4.39	5.18	5.19	4.12	5.74	5.10
Octatetraene	2 ^1A_g	4.16	2.50	2.44	4.84	3.00	3.40	10.18	3.27	4.32	6.50	4.35	5.31	4.47
	1 ^1B_u	4.24	3.36	2.62	4.29	3.94	3.55	9.24	4.19	4.53	4.66	3.51	5.13	4.66
Acetone	1 $^1A_2$	3.96	5.22	4.66	4.52	5.32	5.06	11.61	5.41	5.16	6.15	5.77	5.68	4.40
	2 $^1A_1$	8.44	6.22	8.43	9.48	6.00	11.52	15.58	10.58	11.12	11.46	9.64	11.88	9.40
	1 $^1B_1$	7.88	8.20	8.30	8.70	8.90	9.13	15.65	7.64	10.10	10.90	8.46	10.90	9.10
Formaldehyde	1 $^1A_2$	3.52	4.72	4.20	3.96	4.82	4.57	11.35	4.83	4.60	4.63	5.46	5.11	3.88
	1 $^1B_1$	8.21	10.15	8.83	8.84	10.28	9.66	16.38	10.31	9.74	9.93	10.82	10.30	9.10
	2 $^1A_1$	10.27	8.18	3.80	10.32	8.02	4.15	17.90	6.25	10.84	8.66	6.62	10.33	9.30
Formamide	1 $^1A''$	5.23	6.85	6.14	5.69	6.93	6.48	12.88	6.90	6.51	6.78	7.58	7.18	5.63
	2 $^1A'$	8.23	4.92	6.04	8.60	5.91	6.28	15.37	6.55	8.75	8.34	5.09	7.45	7.39
	3 $^1A'$	10.49	7.09	8.26	10.91	6.96	8.07	19.00	7.70	4.96	11.89	7.98	10.34	
Adenine	2 $^1A'$	4.97	3.37	3.63	5.32	3.43	4.71	11.12	3.39	4.85	6.36	3.48	5.14	5.25
	3 $^1A'$	4.61	3.41	4.80	5.07	4.17	5.57	10.73	4.62	6.07	6.07	4.31	7.02	5.25
	4 $^1A'$	5.84	3.73	4.20	6.50	4.17	4.74	12.37	4.51	5.30	8.89	5.22	6.01	
	1 $^1A''$	4.21	4.98	4.75	5.02	5.27	5.60	11.14	6.43	6.22	7.35	5.89	7.95	5.12
	2 $^1A''$	4.90	5.52	5.33	5.67	5.88	6.17	12.00	6.71	6.61	7.86	4.95	6.77	5.75
Benzene	1 $^1B_{1u}$	5.85	4.03	5.08	5.95	3.91	6.28	12.04	3.72	6.27	5.74	2.97	5.31	6.54
	1 $^1B_{2u}$	5.13	3.13	5.14	5.42	3.10	5.34	11.29	2.95	6.31	5.64	3.17	5.56	5.08
	1 $^1E_{1u}$	7.30	4.86	4.93	7.57	5.05	5.63	13.67	5.25	5.60	7.87	5.42	5.40	7.13
	1 $^1E_{2g}$	8.22	5.64	6.01	9.04	6.76	8.38	15.36	6.54	7.18	10.22	6.97	6.66	8.41
Naphthalene	2 ^1A_g	5.76	3.50	5.11	6.21	3.68	5.72	11.92	3.95	5.86	7.05	3.82	5.85	5.87
	3 ^1A_g	6.14	3.74	3.27	6.86	4.05	4.12	13.00	4.91	6.96	9.20	5.94	4.53	6.67
	1 $^1B_{2u}$	4.05	3.11	3.84	4.29	3.30	3.45	9.71	3.48	4.39	4.76	2.72	3.93	4.77
	2 $^1B_{2u}$	5.84	4.24	4.14	6.17	4.41	5.06	11.76	4.87	5.53	7.13	4.70	4.67	6.33
	3 $^1B_{2u}$	7.67	5.42	5.46	8.17	5.96	6.82	14.30	6.57	9.91	9.32	7.10	10.61	
	1 $^1B_{3u}$	4.15	2.51	3.42	4.41	2.53	4.46	9.81	2.50	4.66	5.10	2.40	4.32	4.24
	2 $^1B_{3u}$	6.09	3.75	3.24	6.38	4.09	4.31	11.73	4.38	4.44	7.10	4.71	4.24	6.06
	3 $^1B_{3u}$	7.65	4.53	4.76	8.66	5.13	5.71	14.68	5.90	6.32	9.64	6.76	8.52	
	1 $^1B_{1g}$	5.01	2.97	3.67	5.60	3.45	5.07	11.10	4.05	5.48	6.29	4.06	5.50	5.99
	2 $^1B_{1g}$	6.20	4.07	3.05	6.41	4.20	4.05	12.43	4.23	4.43	7.75	4.78	4.83	6.47
MSD		-0.32	-1.52	-1.54	0.11	-1.23	-0.70	6.26	-0.93	0.18	0.92	-0.88	0.30	
MAD		0.48	1.77	1.43	0.32	1.52	1.00	6.26	1.44	0.77	1.11	1.40	1.17	
MSD [x-cDFT]		-0.25	-0.53	-0.76	0.04	-0.31	-0.03	6.29	-0.27	0.56	0.59	-0.07	0.70	
MAD [x-cDFT]		0.34	1.10	0.99	0.16	0.98	0.66	6.29	1.18	0.59	0.88	1.21	0.70	

Table S2: Benchmark of singlet excitations energies [eV] for standard and scaled COOX at PBE/def2-TZVP level of theory.

Molecule	State	TDA	COOX$^{(0)}$			COOX			TBE
			ΔE	%T_1	%T_2	ΔE	%T_1	%T_2	
Ethylene	$1\ ^1B_{1u}$	8.42	6.38	90.5%	9.5%	5.51	94.7%	0.0%	7.80
Butadiene	$2\ ^1A_g$	6.31	8.11	0.0%	100.0%	4.34	91.4%	8.6%	6.55
	$1\ ^1B_u$	6.18	4.47	88.4%	11.6%	3.66	91.5%	0.0%	6.18
Hexatriene	$2\ ^1A_g$	5.08	6.23	0.0%	100.0%	3.03	98.6%	0.0%	5.09
	$1\ ^1B_u$	5.07	3.39	90.3%	9.7%	2.87	92.9%	0.0%	5.10
Octatetraene	$2\ ^1A_g$	4.20	5.11	0.0%	100.0%	2.46	97.6%	0.0%	4.47
	$1\ ^1B_u$	4.35	2.76	91.1%	8.9%	2.37	93.6%	0.0%	4.66
Acetone	$1\ ^1A_2$	4.21	4.67	98.2%	1.8%	4.65	98.4%	1.6%	4.40
	$2\ ^1A_1$	8.49	9.97	58.6%	41.4%	9.39	76.2%	23.8%	9.40
	$1\ ^1B_1$	8.13	8.74	84.6%	15.4%	7.90	82.9%	17.1%	9.10
Formaldehyde	$1\ ^1A_2$	3.80	4.20	98.6%	1.4%	4.20	98.7%	1.3%	3.88
	$1\ ^1B_1$	8.82	8.84	66.2%	33.8%	8.78	65.8%	34.2%	9.10
	$2\ ^1A_1$	10.47	10.16	13.3%	86.7%	3.73	91.1%	8.9%	9.30
Formamide	$1\ ^1A''$	5.45	6.16	96.9%	3.1%	6.13	97.3%	2.7%	5.63
	$2\ ^1A'$	8.54	14.02	0.0%	100.0%	5.91	100.0%	0.0%	7.39
	$3\ ^1A'$	10.64	13.20	62.3%	37.7%	9.92	93.2%	0.0%	
Adenine	$2\ ^1A'$	5.10	5.72	67.8%	32.2%	3.64	84.4%	0.0%	5.25
	$3\ ^1A'$	4.69	4.87	83.4%	16.6%	3.95	95.4%	0.0%	5.25
	$4\ ^1A'$	5.91	8.80	43.4%	56.6%	3.81	93.4%	0.0%	
	$1\ ^1A''$	4.28	4.79	96.6%	3.4%	4.77	97.1%	2.9%	5.12
	$2\ ^1A''$	5.02	5.38	97.9%	2.1%	5.34	98.4%	1.6%	5.75
Benzene	$1\ ^1B_{1u}$	6.27	10.79	0.0%	100.0%	4.95	96.2%	0.0%	6.54
	$1\ ^1B_{2u}$	5.29	10.34	0.0%	100.0%	5.13	99.8%	0.0%	5.08
	$1\ ^1E_{1u}$	7.54	11.27	0.0%	100.0%	4.82	93.2%	0.0%	7.13
	$1\ ^1E_{2g}$	8.32	10.61	24.2%	75.8%	5.30	93.3%	0.0%	8.41
Naphthalene	$2\ ^1A_g$	5.85	9.87	19.2%	80.8%	4.96	85.7%	0.0%	5.87
	$3\ ^1A_g$	6.24	9.36	38.2%	61.8%	3.30	96.5%	0.0%	6.67
	$1\ ^1B_{2u}$	4.25	3.98	83.8%	16.2%	2.97	87.1%	0.0%	4.77
	$2\ ^1B_{2u}$	6.12	6.09	76.4%	23.6%	4.11	83.5%	0.0%	6.33
	$3\ ^1B_{2u}$	7.89	8.79	68.3%	31.7%	5.26	84.5%	0.0%	
	$1\ ^1B_{3u}$	4.23	6.95	0.0%	100.0%	3.44	99.3%	0.7%	4.24
	$2\ ^1B_{3u}$	6.23	7.97	0.0%	100.0%	3.11	91.0%	0.0%	6.06
	$3\ ^1B_{3u}$	8.02	19.07	0.0%	[1]150.0%	3.32	97.2%	0.0%	
	$1\ ^1B_{1g}$	5.03	6.91	0.0%	100.0%	3.74	90.7%	9.3%	5.99
	$2\ ^1B_{1g}$	6.49	8.14	0.0%	100.0%	2.98	87.3%	0.0%	6.47

[1] 3-electron excitation

Table S3: Benchmark of singlet excitations energies [eV] for standard and scaled COOX at PBE0/def2-TZVP level of theory.

Molecule	State	TDA	COOX$^{(0)}$			COOX			TBE
			ΔE	%T_1	%T_2	ΔE	%T_1	%T_2	
Ethylene	$1\ ^1B_{1u}$	8.42	7.55	91.7%	8.3%	6.71	94.5%	0.0%	7.80
Butadiene	$2\ ^1A_g$	6.31	8.96	0.0%	100.0%	5.97	81.4%	18.6%	6.55
	$1\ ^1B_u$	6.18	5.46	93.0%	7.0%	4.92	94.7%	0.0%	6.18
Hexatriene	$2\ ^1A_g$	5.08	7.21	0.0%	100.0%	4.11	99.3%	0.0%	5.09
	$1\ ^1B_u$	5.07	4.32	94.7%	5.3%	4.00	96.1%	0.0%	5.10
Octatetraene	$2\ ^1A_g$	4.20	6.04	0.0%	100.0%	3.42	97.3%	0.0%	4.47
	$1\ ^1B_u$	4.35	3.64	95.6%	4.4%	3.42	97.1%	0.0%	4.66
Acetone	$1\ ^1A_2$	4.21	6.12	97.9%	2.1%	5.06	98.2%	1.8%	4.40
	$2\ ^1A_1$	8.49	11.53	0.0%	100.0%	9.39	92.4%	0.0%	9.40
	$1\ ^1B_1$	8.13	9.20	97.9%	2.1%	9.04	99.6%	0.4%	9.10
Formaldehyde	$1\ ^1A_2$	3.80	4.58	98.5%	1.5%	4.57	98.6%	1.4%	3.88
	$1\ ^1B_1$	8.82	9.64	96.9%	3.1%	9.61	97.3%	2.7%	9.10
	$2\ ^1A_1$	10.47	11.51	0.0%	100.0%	4.64	97.2%	2.8%	9.30
Formamide	$1\ ^1A''$	5.45	6.49	97.2%	2.8%	6.47	97.4%	2.6%	5.63
	$2\ ^1A'$	8.54	10.81	52.3%	47.7%	6.06	82.1%	0.0%	7.39
	$3\ ^1A'$	10.64	21.97	0.0%	100.0%	7.71	80.4%	19.6%	
Adenine	$2\ ^1A'$	5.10	6.89	67.1%	32.9%	4.64	84.3%	0.0%	5.25
	$3\ ^1A'$	4.69	5.78	83.9%	16.1%	4.66	92.0%	0.0%	5.25
	$4\ ^1A'$	5.91	10.04	39.4%	60.6%	4.61	90.4%	0.0%	
	$1\ ^1A''$	4.28	5.62	97.3%	2.7%	5.53	98.8%	1.2%	5.12
	$2\ ^1A''$	5.02	6.19	97.7%	2.3%	6.11	99.1%	0.9%	5.75
Benzene	$1\ ^1B_{1u}$	6.27	11.49	0.0%	100.0%	6.17	97.7%	0.0%	6.54
	$1\ ^1B_{2u}$	5.29	11.13	0.0%	100.0%	6.28	99.9%	0.0%	5.08
	$1\ ^1E_{1u}$	7.54	12.05	0.0%	100.0%	5.47	92.8%	0.0%	7.13
	$1\ ^1E_{2g}$	8.32	10.71	81.2%	18.8%	7.73	73.2%	26.8%	8.41
Naphthalene	$2\ ^1A_g$	5.85	11.82	14.8%	85.2%	5.69	90.1%	0.0%	5.87
	$3\ ^1A_g$	6.24	12.56	17.5%	82.5%	4.09	90.9%	0.0%	6.67
	$1\ ^1B_{2u}$	4.25	4.96	86.2%	13.8%	4.02	89.0%	0.0%	4.77
	$2\ ^1B_{2u}$	6.12	7.18	77.2%	22.8%	5.09	83.5%	0.0%	6.33
	$3\ ^1B_{2u}$	7.89	9.34	78.1%	21.9%	6.77	86.9%	0.0%	
	$1\ ^1B_{3u}$	4.23	7.72	0.0%	100.0%	4.46	99.4%	0.6%	4.24
	$2\ ^1B_{3u}$	6.23	8.62	0.0%	100.0%	4.20	93.6%	0.0%	6.06
	$3\ ^1B_{3u}$	8.02	16.65	0.0%	100.0%	4.00	88.6%	0.0%	
	$1\ ^1B_{1g}$	5.03	7.70	0.0%	100.0%	5.05	85.6%	0.0%	5.99
	$2\ ^1B_{1g}$	6.49	8.75	0.0%	100.0%	4.03	89.3%	0.0%	6.47

Table S4: Benchmark of singlet excitations energies [eV] for standard and scaled COOX at ωB97-X/def2-TZVP level of theory.

Molecule	State	TDA	COOX$^{(0)}$			COOX			TBE
			ΔE	%T_1	%T_2	ΔE	%T_1	%T_2	
Ethylene	$1\,^1B_{1u}$	8.26	8.59	88.3%	11.7%	7.70	97.5%	0.0%	7.80
Butadiene	$2\,^1A_g$	7.83	10.51	71.3%	28.7%	7.26	87.7%	0.0%	6.55
	$1\,^1B_u$	6.39	6.28	91.4%	8.6%	5.81	99.0%	0.0%	6.18
Hexatriene	$2\,^1A_g$	7.63	9.81	0.0%	100.0%	4.59	92.0%	0.0%	5.09
	$1\,^1B_u$	5.35	5.25	92.3%	7.7%	4.88	98.6%	0.0%	5.10
Octatetraene	$2\,^1A_g$	6.39	7.82	0.0%	100.0%	4.28	97.5%	0.0%	4.47
	$1\,^1B_u$	4.67	4.62	92.1%	7.9%	4.27	98.1%	0.0%	4.66
Acetone	$1\,^1A_2$	4.47	5.16	97.9%	2.1%	5.15	98.1%	1.9%	4.40
	$2\,^1A_1$	10.15	14.34	54.7%	45.3%	8.33	84.6%	0.0%	9.40
	$1\,^1B_1$	9.00	9.50	97.7%	2.3%	9.39	98.8%	1.2%	9.10
Formaldehyde	$1\,^1A_2$	3.94	4.60	98.4%	1.6%	4.60	98.5%	1.5%	3.88
	$1\,^1B_1$	9.12	9.70	97.1%	2.9%	9.67	97.4%	2.6%	9.10
	$2\,^1A_1$	9.88	12.20	46.9%	53.1%	4.26	94.4%	0.0%	9.30
Formamide	$1\,^1A''$	5.65	6.52	97.3%	2.7%	6.50	97.5%	2.5%	5.63
	$2\,^1A'$	7.96	13.36	26.9%	73.1%	6.69	88.1%	0.0%	7.39
	$3\,^1A'$	12.17	14.42	79.2%	20.8%	11.04	92.7%	0.0%	
Adenine	$2\,^1A'$	5.62	8.79	42.5%	57.5%	4.89	87.4%	0.0%	5.25
	$3\,^1A'$	5.69	6.19	85.4%	14.6%	5.13	94.0%	0.0%	5.25
	$4\,^1A'$	7.40	12.66	28.6%	71.4%	4.96	91.2%	0.0%	
	$1\,^1A''$	5.53	6.22	95.2%	4.8%	5.91	99.8%	0.0%	5.12
	$2\,^1A''$	6.14	6.78	95.3%	4.7%	6.41	99.2%	0.0%	5.75
Benzene	$1\,^1B_{1u}$	8.48	12.47	0.0%	100.0%	5.98	97.1%	0.0%	6.54
	$1\,^1B_{2u}$	6.61	12.04	0.0%	100.0%	6.19	99.4%	0.6%	5.08
	$1\,^1E_{1u}$	7.79	12.93	0.0%	100.0%	5.37	95.8%	0.0%	7.13
	$1\,^1E_{2g}$	9.93	10.52	93.2%	6.8%	9.72	98.4%	0.0%	8.41
Naphthalene	$2\,^1A_g$	6.63	13.51	0.9%	99.1%	5.53	85.7%	0.0%	5.87
	$3\,^1A_g$	8.70	14.29	26.2%	73.8%	4.18	90.3%	0.0%	6.67
	$1\,^1B_{2u}$	4.98	5.58	77.0%	23.0%	4.24	91.2%	0.0%	4.77
	$2\,^1B_{2u}$	6.86	7.85	66.0%	34.0%	5.15	86.7%	0.0%	6.33
	$3\,^1B_{2u}$	8.85	9.49	87.7%	12.3%	7.97	93.9%	0.0%	
	$1\,^1B_{3u}$	4.72	8.66	0.0%	100.0%	4.55	99.3%	0.7%	4.24
	$2\,^1B_{3u}$	6.64	9.24	0.0%	100.0%	4.38	96.8%	0.0%	6.06
	$3\,^1B_{3u}$	9.65	17.82	11.6%	88.4%	5.04	90.2%	9.8%	
	$1\,^1B_{1g}$	6.56	6.72	94.3%	5.7%	6.32	99.1%	0.0%	5.99
	$2\,^1B_{1g}$	6.98	7.08	93.9%	6.1%	6.63	98.8%	0.0%	6.47

Table S5: Benchmark of singlet excitations energies [eV] for standard and scaled COOX at HF/def2-TZVP level of theory.

Molecule	State	TDA	COOX$^{(0)}$			COOX			TBE
			ΔE	%T_1	%T_2	ΔE	%T_1	%T_2	
Ethylene	1 $^1B_{1u}$	7.97	8.28	89.1%	10.9%	7.50	99.3%	0.0%	7.80
Butadiene	2 ^1A_g	8.32	9.82	77.9%	22.1%	7.19	88.6%	0.0%	6.55
	1 ^1B_u	6.40	6.57	90.8%	9.2%	5.93	97.6%	0.0%	6.18
Hexatriene	2 ^1A_g	7.69	11.06	0.0%	100.0%	5.40	94.8%	5.2%	5.09
	1 ^1B_u	5.43	5.65	89.3%	10.7%	5.03	97.4%	0.0%	5.10
Octatetraene	2 ^1A_g	6.93	9.85	0.0%	100.0%	4.26	93.5%	0.0%	4.47
	1 ^1B_u	4.79	5.04	87.6%	12.4%	4.37	96.4%	0.0%	4.66
Acetone	1 $^1A_2$	5.15	5.67	97.4%	2.6%	5.61	98.3%	1.7%	4.40
	2 $^1A_1$	12.49	14.62	79.8%	20.2%	12.28	99.5%	0.5%	9.40
	1 $^1B_1$	9.79	10.22	97.2%	2.8%	10.05	98.8%	1.2%	9.10
Formaldehyde	1 $^1A_2$	4.51	5.04	97.8%	2.2%	9.97	96.5%	3.5%	3.88
	1 $^1B_1$	9.65	10.10	97.5%	2.5%	10.03	98.1%	1.9%	9.10
	2 $^1A_1$	9.63	10.10	84.3%	15.7%	8.02	91.8%	0.0%	9.30
Formamide	1 $^1A''$	6.49	7.07	97.3%	2.7%	7.03	97.9%	2.1%	5.63
	2 $^1A'$	12.32	16.69	0.0%	100.0%	6.32	92.2%	0.0%	7.39
	3 $^1A'$	13.87	23.70	6.9%	93.1%	10.92	82.1%	0.0%	
Adenine	2 $^1A'$	6.31	9.05	54.9%	45.1%	5.07	85.3%	0.0%	5.25
	3 $^1A'$	6.18	7.06	81.2%	18.8%	5.42	91.4%	0.0%	5.25
	4 $^1A'$	8.97	13.11	28.9%	71.1%	5.94	99.4%	0.6%	
	1 $^1A''$	7.04	7.85	90.8%	9.2%	6.97	97.4%	0.0%	5.12
	2 $^1A''$	7.50	9.56	75.1%	24.9%	6.78	88.7%	0.0%	5.75
Benzene	1 $^1B_{1u}$	6.20	13.23	0.0%	100.0%	5.45	99.8%	0.2%	6.54
	1 $^1B_{2u}$	6.05	13.72	0.0%	100.0%	5.31	98.9%	1.1%	5.08
	1 $^1E_{1u}$	8.09	13.02	0.0%	100.0%	5.14	95.4%	0.0%	7.13
	1 $^1E_{2g}$	10.62	13.41	73.0%	27.0%	9.01	84.6%	0.0%	8.41
Naphthalene	2 ^1A_g	7.27	13.16	28.5%	71.5%	5.92	80.6%	0.0%	5.87
	3 ^1A_g	9.40	13.19	57.6%	42.4%	4.80	94.5%	5.5%	6.67
	1 $^1B_{2u}$	5.02	6.51	57.2%	42.8%	3.94	88.7%	0.0%	4.77
	2 $^1B_{2u}$	7.20	8.89	45.6%	54.4%	4.65	89.1%	0.0%	6.33
	3 $^1B_{2u}$	9.61	10.24	84.5%	15.5%	8.54	97.1%	0.0%	
	1 $^1B_{3u}$	5.18	9.74	0.0%	100.0%	4.31	97.6%	2.4%	4.24
	2 $^1B_{3u}$	7.04	10.11	0.0%	100.0%	4.15	98.7%	0.0%	6.06
	3 $^1B_{3u}$	12.10	22.08	0.3%	99.7%	5.31	79.2%	0.0%	
	1 $^1B_{1g}$	6.72	9.90	51.6%	48.4%	5.43	80.6%	0.0%	5.99
	2 $^1B_{1g}$	7.87	10.39	54.1%	45.9%	4.85	92.8%	7.2%	6.47
